# Single-cell RNA analysis identifies pre-migratory neural crest cells expressing markers of differentiated derivatives

**DOI:** 10.7554/eLife.66078

**Published:** 2021-08-16

**Authors:** Ezra Lencer, Rytis Prekeris, Kristin Bruk Artinger

**Affiliations:** 1 Department of Craniofacial Biology, University of Colorado Denver United States; 2 Department of Cell and Developmental Biology, University of Colorado Denver United States; Washington University School of Medicine United States; Memorial Sloan Kettering Cancer Center United States

**Keywords:** rohon-beard cells, xanthophores, pigment development, neural crest cells, scRNA-seq, Zebrafish

## Abstract

The neural crest is a migratory population of stem-like cells that contribute to multiple traits including the bones of the skull, peripheral nervous system, and pigment. How neural crest cells differentiate into diverse cell types is a fundamental question in the study of vertebrate biology. Here, we use single-cell RNA sequencing to characterize transcriptional changes associated with neural crest cell development in the zebrafish trunk during the early stages of migration. We show that neural crest cells are transcriptionally diverse and identify pre-migratory populations already expressing genes associated with differentiated derivatives, specifically in the xanthophore lineage. Further, we identify a population of Rohon–Beard neurons in the data. The data presented identify novel genetic markers for multiple trunk neural crest cell populations and Rohon–Beard neurons providing insight into previously uncharacterized genes critical for vertebrate development.

## Introduction

A fundamental question in developmental biology concerns how multipotent cell precursors produce diverse cell types (Waddington, 1957). This question is particularly relevant for the study of the vertebrate neural crest, a transient population of stem-like cells that contribute to multiple traits and form numerous cell types. Neural crest cells (NCCs) make the cartilages and bones of the skull, neurons of the peripheral nervous system (PNS), glial cells, and pigment cells among other derivatives (Anderson, 1989; Theveneau and Mayor, 2012). How and when NCCs acquire these diverse fates are long-standing questions in the study of vertebrate development and have direct implications for both our understanding of vertebrate evolution as well as the origins of NCC-derived hereditary diseases such as cleft lip and palate.

NCC development is classically conceived as a stepwise series of bifurcating cell fate decisions (Anderson, 1989; Klymkowsky et al., 2010; Simoes-Costa et al., 2014). NCCs are specified at the neural plate border, a region of ectoderm in the gastrula stage embryo that also produces cranial placodes, and in fishes and frogs, Rohon–Beard sensory neurons. Once specified, NCCs undergo an epithelial-to-mesenchymal transition, migrate throughout the embryo, and differentiate into multiple cell types. When and how NCCs differentiate into these derivative lineages remains unclear. Early studies in chick and frog suggested that migratory NCCs are multipotent, requiring cues from the migratory environment to initiate differentiation (Bronner-Fraser and Fraser, 1988; Bronner-Fraser and Fraser, 1989; Collazo et al., 1993). Other studies in zebrafish and quail suggested that NCCs are often lineage restricted prior to migration, and thus interactions among NCCs in the neural tube must somehow specify cells to different lineages before cells become migratory (Henion and Weston, 1997; Raible and Eisen, 1994; Raible and Eisen, 1996; Schilling and Kimmel, 1994).

Current understanding of the transcriptional changes associated with different stages of NCC development has largely been built from analysis of gene expression in whole tissues (Simoes-Costa et al., 2014). However, developing tissues are often transcriptionally heterogeneous, a fact highlighted by recent developments in single-cell RNA sequencing (scRNA-seq) (Briggs et al., 2018; Farnsworth et al., 2020; Farrell et al., 2018; Morrison et al., 2020; Morrison et al., 2017; Saunders et al., 2019; Soldatov et al., 2019; Wagner et al., 2018; Zalc et al., 2021). Furthermore, as cells within tissues are often at different stages of fate specification, scRNA-seq has the potential to expose transcriptional changes associated with differentiation that would have been missed by bulk sequencing experiments (Farrell et al., 2018; Soldatov et al., 2019). For instance, in chick, scRNA-seq studies of migrating cranial NCCs identified unique transcriptional signatures associated with a morphologically cryptic population of cells at the leading migratory edge (Morrison et al., 2020; Morrison et al., 2017). Thus, an emerging picture is that NCCs are more transcriptionally heterogeneous than once thought, and that a complete understanding of NCC biology must account for this heterogeneity.

**Figure 1.**
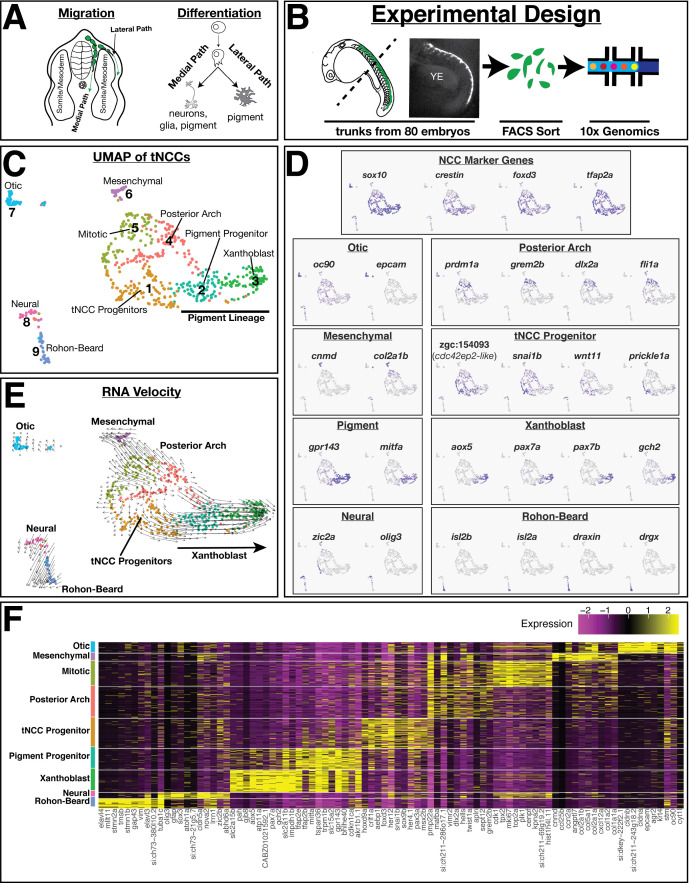
Single-cell RNA analysis identifies transcriptionally heterogeneous *sox10*:eGFP population. (**A**) Schematic of zebrafish trunk neural crest cell (tNCC) migration. Medial route migrating tNCCs produce peripheral nervous system and pigment cells, while tNCCs migrating along the dorsal-lateral path produce pigment cells. (**B**) Schematic of experimental design. NCCs were sampled by dissecting the trunks of 80 *sox10*:eGFP+ zebrafish at 24 hr post fertilization. GFP+ NCCs were FAC sorted and sequenced using 10X Genomics. Representative image shows *sox10*:eGFP transgene in a dissected trunk used for cell dissociation. YE: yolk extension. (**C**) Uniform Manifold Approximation and Projection (UMAP) and clustering of cells reveals multiple transcriptionally unique *sox10*:eGFP+ cell clusters. Numbers and labels correspond to main text. (**D**) Expression of select genes in UMAP space shows variable expression across different clusters. (**E**) RNA velocity connects cells in developmental pseudotime. Direction and length of arrows represent predicted direction of differentiation as calculated by Velocyto (see Materials and methods). Note cells predicted to be differentiating from tNCC progenitor cluster one into xanthoblast cluster 3. (**F**) Heatmap shows expression of top 10 most differentially expressed genes (log2Fold) for each cluster. Figure 1—source code 1.Annotated text file of R code used to generate analyses presented in Figures 1 and 2.Data deposited in NCBI GEO GSE112294.

Here, we use scRNA-seq to characterize the transcriptional landscape of trunk neural crest cells (tNCCs) during the early stages of migration in zebrafish. We sampled NCCs at a stage in zebrafish development when both migratory and pre-migratory tNCC populations are present in the embryo. In the trunk, NCCs migrate along one of two paths that are temporally and spatially separated. Early migrating tNCCs move between the neural tube and somitic mesoderm along the medial path (Figure 1A). These cells give rise to neurons of the PNS, glia, and pigment cells. Later migrating tNCCs follow the dorsolateral path between the skin ectoderm and somitic mesoderm to produce pigment cells. Thus, our experiment aims to capture transcriptional changes associated with the onset of migration and the splitting of NCCs into a neuronal or pigment lineage. We show that tNCCs are a transcriptionally diverse population of cells, and that some pre-migratory tNCCs are already expressing genes associated with a differentiated xanthophore pigment lineage. In addition, we identify a unique population of Rohon–Beard neurons (RBs) that expresses *sox10* and comment on the developmental similarity among RBs and NCCs. Ultimately these data create a foundation for future genetic work studying the roles of previously uncharacterized genes during NCC and RB development.

**Figure 1—figure supplement 1.**
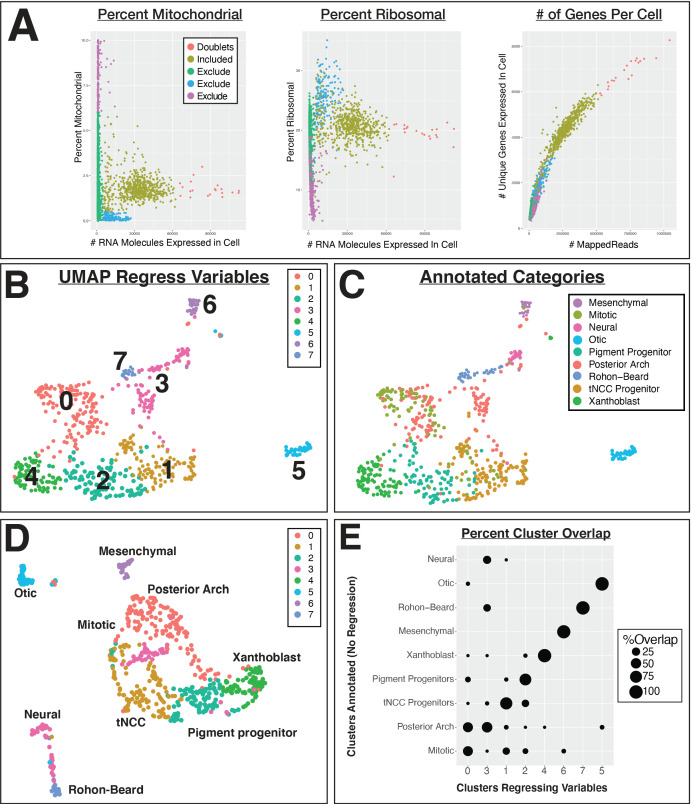
Analysis of single-cell RNA sequencing (scRNA-seq) is robust to different analysis parameters. (**A**) Scatterplots show quality filtering of scRNA-seq data. Cells were filtered based on percent mitochondrial expression and number of RNA molecules (UMI) expressed by each cell. Cells labeled ‘included’ were used for analyses. Colors visualize groups of cells across different scatterplots. (**B–E**) The same biologically relevant groups of cells are identified using different analysis parameters. (**B**) Uniform Manifold Approximation and Projection (UMAP) and clustering of cells after regressing out effects of cell cycle, mitochondrial contribution, and ribosomal contribution in the data. (**C**) Same UMAP as in panel (**B**) with cells labeled by cluster identity as in Figure 1C. (**D**) UMAP from Figure 1C with cells colored as in panel (**B**). Names correspond to annotated clusters in Figure 1C. Note concordance across different analysis parameters. The mitotic cluster cells from Figure 1C are assigned to posterior arch and trunk neural crest cell clusters after regressing out effects of cell cycle. (**E**) The same clusters are obtained using different analysis parameters. Dot size represents percent of cells assigned to each cluster from Figure 1C that contribute to each cluster in panel (**B**). Note that different analysis parameters identify the same cell populations.

**Figure 1—figure supplement 2.**
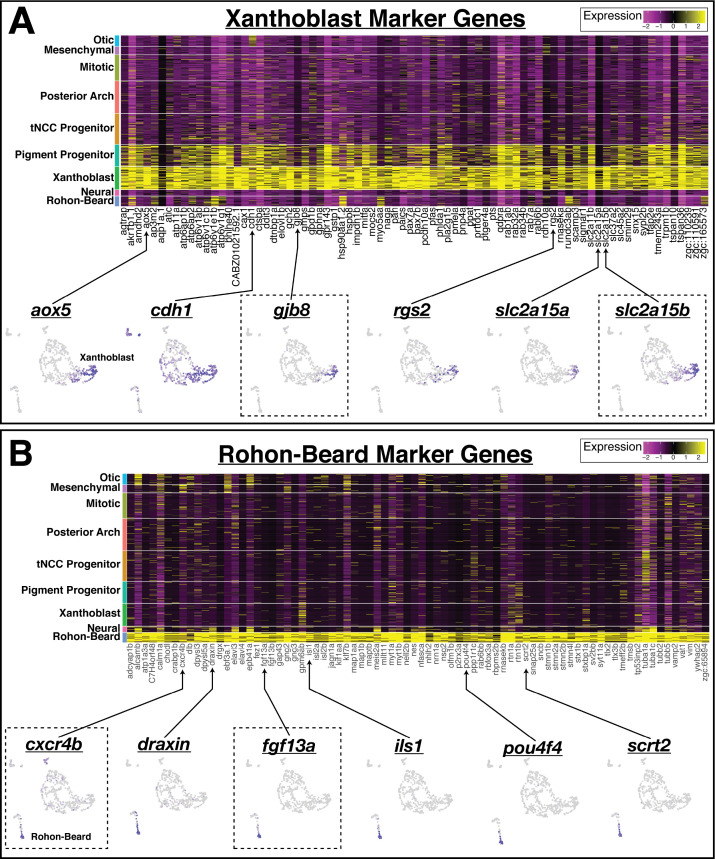
Expression of genes that label xanthoblast and Rohon–Beard clusters. Heatmap shows the top differentially expressed genes (log2Fold) that label the xanthoblast (**A**) and Rohon–Beard (**B**) clusters. Expression of select genes in Uniform Manifold Approximation and Projection space is shown below. Dotted lines box genes shown in Figure 3. For plots, the top 80 most differentially expressed (log2Fold) genes were selected excluding genes without names.

**Figure 1—figure supplement 3.**
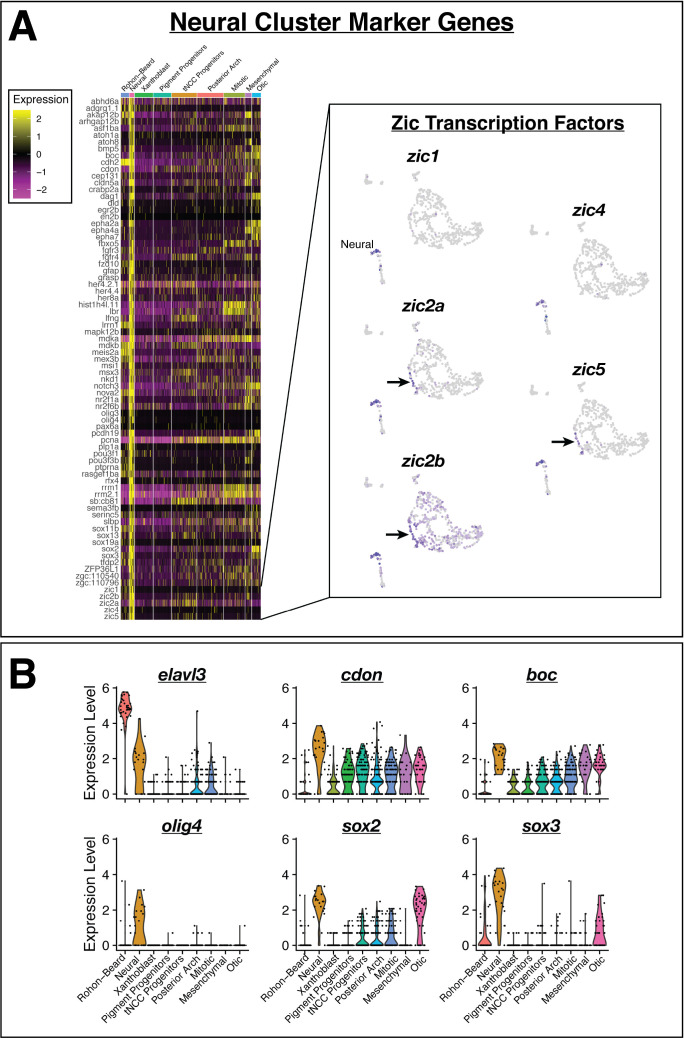
Expression of genes that label neural cluster 8 cells. (**A**) Heatmap of top most differentially expressed genes (log2Fold) expressed by cluster 8 neural cells. Inset shows expression of multiple *zic* transcription factors that label the neural cell cluster in Uniform Manifold Approximation and Projection space. Note that *zic2a/b* and *zic5* are also expressed by a subset of trunk neural crest cell progenitor cells (arrows). (**B**) Violin plots for select genes in cluster 8 identify these cells as possible neural/neural plate cells.

**Figure 1—figure supplement 4.**
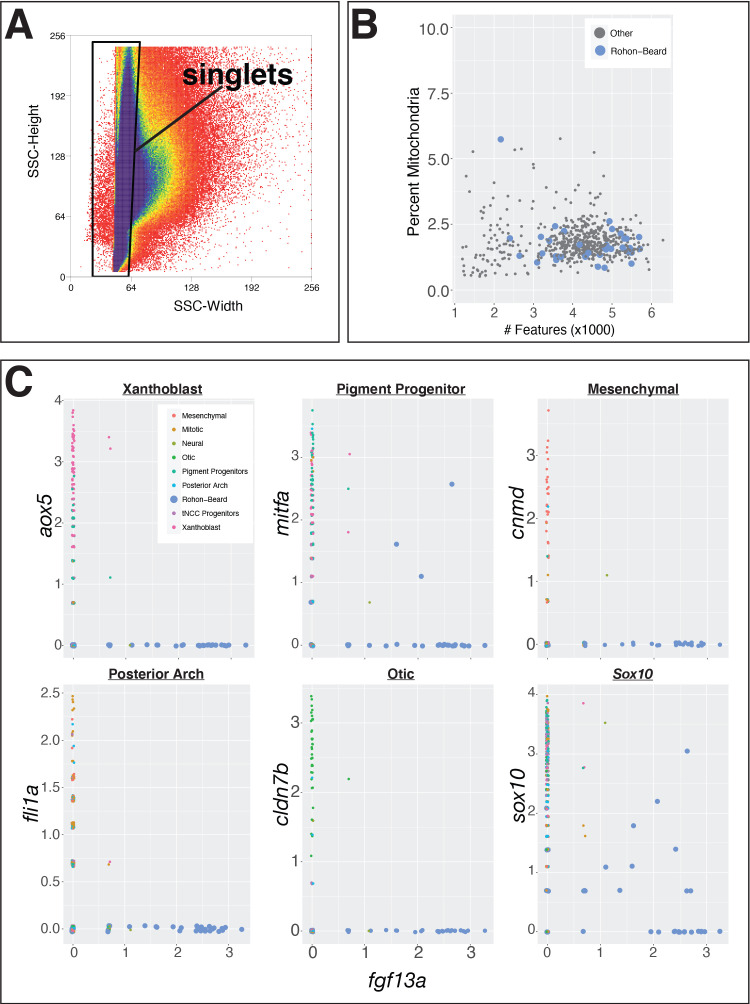
Rohon–Beard cells are unlikely to be doublets from incomplete cell dissociation. (**A**) FAC sorting gated for singlet cells (black box). (**B**) Possible doublets were quality filtered prior to analysis by excluding cells with large UMI counts. Rohon–Beard neurons (RBs; red points) passed quality filtering and are mixed with other neural crest cells (NCCs) in quality check plots. (**C**) RBs do not express markers of other NCC clusters, with the exception of three *mitfa* expressing RBs. RBs do express *sox10* at a low level.

## Results

### scRNA-seq identifies transcriptionally unique populations of NCCs

We used 10X Genomics to characterize the transcriptomes of 607 tg(–4.9*sox10*:eGFP)-positive cells from the dissected trunks of 20–24 hr post fertilization (hpf) zebrafish embryos (25 somite stage; hereafter 24 hpf; Figure 1B). At this developmental stage, the *sox10*:eGFP transgene labels NCCs as well as the otic organ and lateral line (Carney et al., 2006). tNCCs in the anterior segments of the body are beginning to migrate along the medial path (Figure 1A and B), while most tNCCs that populate the posterior segments of the body and dorsolateral pathway are pre-migratory. Thus our sample represents the early stages of tNCC migration and includes a mixed population of migratory and pre-migratory tNCCs.

Our scRNA-seq analysis identified a transcriptionally heterogeneous population of sox10:eGFP+ cells (Figure 1C–F). Most cells were assigned to the NCC lineage based on expression of genes known to label NCCs at this stage (*crestin, sox10, foxd3,* and *tfap2a*; Figure 1C and D). We also identified a cluster of cells (cluster 7, 39 cells, Figure 1C) that belongs to the otic organ or lateral line (*epcam*, *cldn7b*, *f11r.1*, *otomp*, *otog*). A small cluster of cells (cluster 6, 28 cells), expressing multiple collagen genes, were assigned to a ‘mesenchymal’ NCC type found in other datasets (Howard et al., 2021; Soldatov et al., 2019), and some cells were assigned as posterior arch cranial NCCs based on the expression of genes known to mark these regions of the embryo (*prdm1a*, *grem2b*, *dlx2a*, and *fli1a*).

These posterior arch cells were split across two clusters (clusters 4 and 5, 116 and 94 cells, respectively), one of which (cluster 5) are proliferating cells expressing genes involved in cell cycle (e.g., *cdk1, mki67, plk1*; Figure 1F). Regressing out cell cycle confirms that most cluster 5 cells are mitotic posterior arch cells, though a small number of these mitotic cells are assigned as tNCCs (Figure 1—figure supplement 1). Importantly, we recover the same biologically relevant groupings of cells and marker genes whether regressing out variables such as cell cycle or not indicating that the biological signal of cell type in the data is robust to different analysis parameters (Figure 1—figure supplement 1; Materials and methods). Since our main interest is on NCCs in the trunk, we do not further analyze the otic, mesenchymal, and posterior arch tissues at length below.

Despite sampling zebrafish embryos during the early stages of tNCC migration, we observed multiple transcriptionally unique populations of tNCCs. One cluster of tNCCs (cluster 1, 112 cells) expresses genes that mark NCCs (e.g., *crestin*, *sox10*, *foxd3*, sox9a, *tfap2a*), and multiple Hox genes (e.g., *hoxb8a*, *hoxa11b*, *hoxb7a*) that place these cells in the posterior section of the zebrafish trunk (Figure 1C, D and F). Cells in cluster 1 express genes associated with tNCC migration (e.g., *snai1b, prickle1a, wnt11*) and genes with unknown function including a predicted cdc42 effector protein (zgc:150493) and cytokine-associated factor *crlf1a*. As tNCCs in this cluster lack expression of genes identifying these cells to a differentiated lineage, we hypothesize cluster 1 as multipotent tNCC progenitors.

We found a large number of tNCCs already expressing genes associated with differentiated pigment cells spread across two clusters (Figure 1C, D and F). This was surprising as these cells are presumably pre-migratory based on the sampled stage. Cells in cluster 2 (79 cells) express melanophore/pan-pigment genes including *mitfa*, *gpr143*, *trpm1b*. We hypothesize these cells as pan-pigment-cell progenitors. In contrast to these pigment progenitors, cells in cluster 3 (82 cells) express genes enriched in differentiated xanthophores (red/orange pigment cells) including *aox5*, *pax7a/b*, and *gch*. We hypothesize cluster 3 as xanthoblast cells (e.g., pre-xanthophore), and these data identify novel markers of the early xanthophore lineage including solute carrier paralogs *slc2a15a/b* and gap junction protein *gjp8* (Figure 1—figure supplement 2).

These data suggest that a subset of tNCCs begin expressing gene regulatory networks (GRNs) associated with pigment cell lineages prior to migration. This raises the possibility that the sequencing data reflect transcriptional profiles of cells at different stages of differentiation. To explore this further, we inferred RNA velocity (Velocyto) as a measure of developmental pseudotime (Bergen et al., 2020). This method uses estimates of RNA splicing to infer how cells may be transitioning across different transcriptional states. RNA velocity showed a strong signal of tNCCs differentiating from NCC progenitor cluster 1 into xanthoblast pigment cluster 3 (Figure 1E) consistent with a hypothesis that some tNCCs initiate a pigment differentiation program prior to migration.

### Identification of neural-like cells and Rohon–Beard neurons

Two clusters (clusters 8 and 9) are distinguished from other cells by expression of genes enriched in the developing spinal cord and differentiated neurons (e.g., *elavl3*, *elavl4*). Cells in cluster 8 (21 cells) express genes associated with the neural plate and neural tube including *olig3*, *olig4*, *sox3*, *boc*, *cdon*, and multiple zic family transcription factors (*zic1*, *zic2a*, *zic2b*, *zic4*, *zic5*, *zic6*). Intriguingly some of these genes (e.g., *zic2a/b*, *zic5*) are also expressed by a subset of tNCC progenitors (Figure 1—figure supplement 3). Cells assigned to this neural cluster may represent ‘early’ NCCs still enriched in genes associated with the neural plate/neural plate border. Alternatively, these cells may be differentiating glia or other neuronal subtype precursors.

Cells in cluster 9 (36 cells) were transcriptionally distinct from all other cells in the dataset and express genes known to mark RBs including *isl1*, *isl2a/b*, *scrt2*, *prdm14*, and *drgx* (Figure 1C, D and F; Figure 1—figure supplement 2). RBs are a non-migratory population of cells in the dorsal neural tube that innervate the skin of embryonic and larval zebrafish. Like NCCs, RBs are also specified at the neural plate border and genes critical for the proper specification of NCCs are also critical for specification of RBs (Artinger et al., 1999; Hernandez-Lagunas et al., 2005; Hernandez-Lagunas et al., 2011). While we cannot fully rule out that RB neurons are represented in our dataset due to the proximity of RBs and NCCs to each other in the neural tube, we find this explanation unlikely. Our FACS sorting gated for doublets, and our bioinformatic pipeline further filtered out putative doublets based (Figure 1—figure supplement 4). With the exception of three *mitfa* expressing cells, RBs do not express genes associated with other NCC clusters, which would be expected if these cells are due to incomplete dissociation from NCC tissue. Instead, our data recovering a putative RB population in an experiment targeting tNCCs may reflect a common developmental origin between these two cell types.

### Cross-dataset integration confirms cell identities and expression of marker genes

To further investigate the transcriptional identities of sox10:eGFP+ cells in our dataset, we integrated our data with an existing scRNA-seq atlas of sox10:eGFP tNCCs from 48 to 50 (hereafter 48 hpf) and 68-70 hpf (hereafter 68 hpf) zebrafish embryos (Howard et al., 2021). These complementary data sampled tNCCs using the same transgene allowing us to directly study tNCC identity across developmental time. For these analyses, we used the Seurat integrate datasets function to combine data from cells isolated from the trunks of 24 hpf, 48 hpf, and 68 hpf zebrafish.

**Figure 2.**
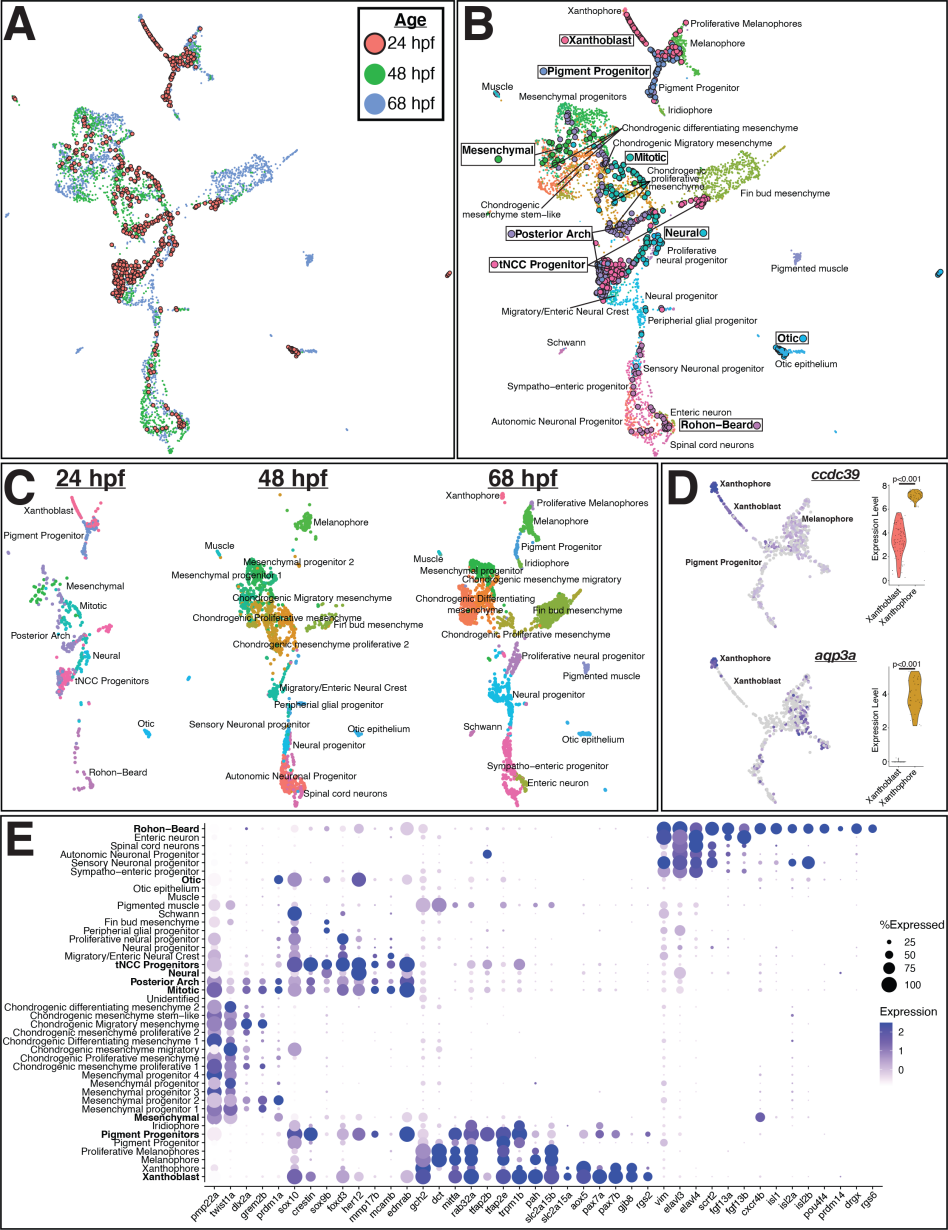
Integrated analysis of *sox10*:eGFP+ cells from 24, 48, and 68 hr post fertilization (hpf) zebrafish embryos shows pre-migratory trunk neural crest cells (tNCCs) expressing markers of derivative cell types. (**A**) Uniform Manifold Approximation and Projection (UMAP) of integrated datasets showing cells colored by age. (**B**) UMAP of integrated datasets with cells colored and labeled by annotated category. Annotations of 48 and 68 hpf cells from Howard et al., 2021 are indicated with regular text. Annotations for 24 hpf cells from the current study are boxed and bolded. Points representing cells from 24hpf embryos are larger and outlined in black for visualization. Note that cells group by cell identity across developmental time. (**C**) Same UMAP as in panel (**B**) with cells split by age. (**D**) Representative genes expressed at higher levels in mature xanthophores from 68 hpf embryos than xanthoblasts from 24 hpf embryos. Expression is shown in UMAP space and as violin plots for the 24 hpf xanthoblast and 68 hpf xanthophore cluster cells. p values are from Wilcoxon rank-sum tests. (**E**) Dotplot of select genes that mark NCCs, pigment progenitors, xanthophores, and Rohon–Beard neurons. Clusters from 24 hpf *sox10*:eGFP+ cells are bolded. Note that xanthoblasts and xanthophores express the same marker genes. Rohon–Beard neurons express a unique set of genes unlike other neural derivatives from older embryos.

Our analysis of these combined datasets found tNCCs clustering by cell type across developmental time (Figure 2). Cells assigned to the otic organ from 24 hpf embryos cluster with cells independently assigned to the otic organ from 48 and 68 hpf embryos. Similarly, mitotic cells from all three datasets group together, as do mesenchymal cell types. Cells assigned as tNCC progenitors at 24 hpf (cluster 1) group proximate to migratory tNCCs from 48 hpf embryos (e.g., ‘Migratory/enteric neural crest’). Posterior arch cells from 24 hpf embryos are split across these NCC progenitor groups and mesenchymal cell types, perhaps reflecting the early developmental stage sampled. Importantly, NCCs from 24 hpf embryos do not group with differentiated neural derivatives from 48 and 68 hpf embryos, suggesting that tNCCs delay initiating a neural differentiation program until post-migratory developmental stages.

**Figure 2—figure supplement 1.**
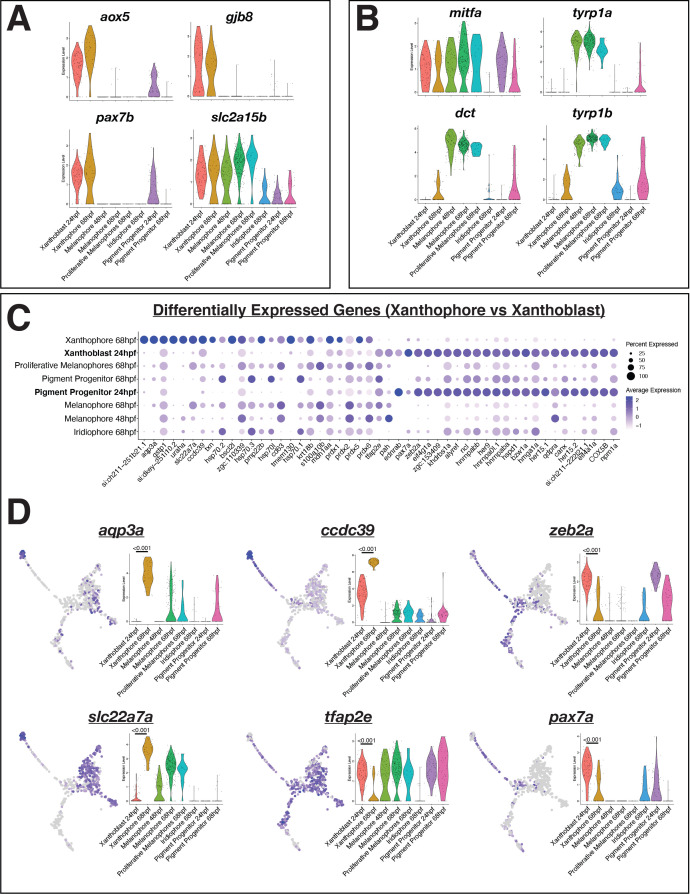
Expression of genes associated with pigment cells over developmental time. (**A**) Violin plots show expression of genes that mark both differentiated xanthophores and pre-migratory xanthoblasts. Note restricted of expression of novel xanthoblast marker gene *gjb8* to the xanthophore lineage, and broader expression of *slc2a15b* to differentiated pigment cells. (**B**) Violin plots show expression of genes associated with melanophore maturation. While cells at all stages express *mitfa*, other genes involved in melanin biosynthesis are expressed at higher levels in 48 and 68 hr post fertilization (hpf) embryos. (**C**) Dotplot of top differentially expressed genes (log2Fold) between 24 hpf xanthoblasts and 68 hpf xanthophores. Note that genes overexpressed in xanthoblasts (right portion of plot) are also expressed by pigment progenitor populations. Cells sampled at 24 hpf are bolded. (**D**) Representative genes differentially expressed between 24 hpf xanthoblasts and 68 hpf xanthophores. For each gene, expression in Uniform Manifold Approximation and Projection space and violin plots shows predicted change in expression over developmental time. p values from Wilcoxon rank-sum tests.

**Figure 2—figure supplement 2.**
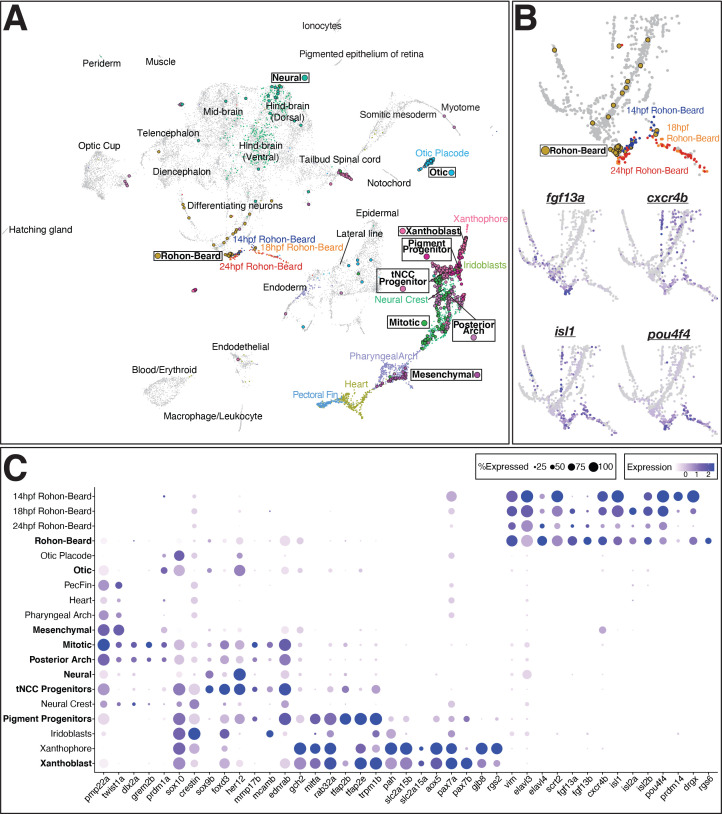
sox10:eGFP+ cells from 24 hr post fertilization (hpf) embryos cluster by cell type on whole embryo zebrafish atlases. (**A**) Uniform Manifold Approximation and Projection of integrated data from 24 hpf *sox10*:eGFP+ cells, and cells from whole zebrafish embryos sampled at 14, 18, and 24 hpf (Wagner et al., 2018). Note that cells group by annotated category across different datasets. Annotations from Wagner et al., 2018 are in regular text. Cells annotated to relevant cell categories for the current study (e.g., neural crest cells [NCCs] and Rohon–Beard neurons [RBs]) are colored. Points representing cells from the current study are larger and outlined in black. Annotations for these sox10:eGFP+ cells are bolded and boxed. Note that cells annotated as NCCs in each dataset group together, as do xanthophore lineage cells. Some NCCs from Wagner et al., 2018 group with hind brain cells (small green points) along with *sox10*:eGFP+ cells annotated to the neural cluster. RBs from all three datasets group together. (**B**) Blowup of RB cell clusters from panel (**A**) and expression of select RB marker genes. (**C**) Dotplot of select genes that label NCCs, pigment cells, and RBs in relevant clusters from panel (**A**). Pigment cells from all datasets express the same genes including novel xanthoblast markers. Similarly, RBs annotated in each dataset express the same genes concordant with identification of an RB gene regulatory networks.

Concordance across tNCCs sampled at different stages is striking for pigment cells (Figure 2). Pigment cell progenitors from 24 hpf embryos group with pigment cell progenitors and melanophores from 48 hpf and 68 hpf embryos (Figure 2B and C). Xanthoblasts sampled from 24 hpf embryos form a bridge connecting this pigment progenitor/melanophore cluster to differentiated xanthophores sampled from 68 hpf embryos. Critically, we find that differentiated xanthophores at 68 hpf and xanthoblasts at 24 hpf express the same set of genes including *gjb8, rgs2, pax7a/b, aox5, slc2a15a/b* (Figure 2E; Figure 2—figure supplement 1). These data further indicate that 24 hpf xanthoblasts are expressing a differentiated xanthophore GRN.

While 24 hpf pigment cells are expressing markers of differentiated derivatives, some differences between early and late pigment cells exist (Figure 2—figure supplement 1). For instance, pigment cells at all developmental stages express critical transcription factors such as *mitfa*, but downstream targets such as *dct* and *tyrp1a/b* that effect melanin biosynthesis are only expressed in cells isolated from 48 hpf and 68 hpf embryos (Figure 2—figure supplement 1). To investigate gene expression changes associated with xanthophore maturation, we looked for differentially expressed genes between 24 hpf xanthoblasts and 68 hpf xanthophores. This test identified genes upregulated in xanthophores at 68 hpf including *aqp3a*, *slc22a7a*, cilia-associated protein *ccdc39*, and *prdx1/2/5/6* genes associated with redox state (Figure 2D; Figure 2—figure supplement 1). Xanthoblasts in contrast express genes associated with EMT and melanophore cell types at higher levels including *tfap2e* and *zeb2a* (Figure 2—figure supplement 1). Thus, these data suggest that there is biological maturation that occurs during this time frame of xanthophore development. Xanthoblasts at 24 hpf retain a pan-pigment transcriptional state while also expressing a mostly complete differentiated xanthophore GRN.

In these analyses, RBs cluster with neuronal cell types (e.g., enteric neurons and spinal cord neurons) sampled from 48 and 68 hpf embryos (Figure 2B). This reflects similar transcriptomes across different neurons. While these other neuronal cells co-express some shared genes with RBs (e.g., *elavl3*, *elavl4*, *vim*), RBs express a unique set of genes (Figure 2E). For instance, RBs and enteric neuron cells from 68 hpf embryos both express *fgf13a*/*b* ligands at appreciable levels. RBs, however, also express *cxcr4b* and islet genes that are lacking in enteric neurons. Sensory neuronal progenitor cells (potential dorsal root ganglia) sampled at 48 hpf express islet genes, but these same cells lack expression of other RB genes such as *pou4f4* and express *cxcr4b* and *fgf13a/b* at very low levels (Figure 2E).

To further investigate cell types in our dataset, and RB cell identity, we next integrated our data with a scRNA-seq atlas of whole zebrafish embryos sampled at 14 hpf, 18 hpf, and 24 hpf (Wagner et al., 2018). Both NCCs and RBs are annotated in these whole embryo datasets. As expected, NCCs from our sox10:eGFP dataset grouped with annotated NCCs sampled from whole embryos at these other stages. We again find that cells assigned to the xanthoblast cluster group with annotated ‘xanthophores’ from these other data. RBs isolated from sox10:eGFP cells cluster with cells annotated as RBs in these other datasets (Figure 2—figure supplement 2). Furthermore, we recover a putative RB GRN across datasets and time points. RBs in all four datasets predict restricted expression of a unique RB set of genes that includes *cxcr4b*, *fgf13a/b*, *isl1/2a/2b*, *prdm14*, and *pou4f4*.

### In situ hybridization confirms novel genetic markers in xanthoblast and RB lineages

Single-cell transcriptomes identify previously uncharacterized genes that are uniquely expressed in pre-migratory xanthoblast and RB cell populations at 24 hpf. We sought to confirm expression of some novel genes predicted to these cell populations using fluorescent quantitative hybridization chain reaction (qHCR) in situ hybridization (Choi et al., 2018).

### *slc2a15b* and *gjb8* are co-expressed in pre-migratory xanthoblast tNCCs

In addition to known markers of the xanthophore lineage, scRNA-seq identified *slc2a15b* and *gjb8* as among the most highly expressed and restricted to the xanthoblast lineage cluster (Figure 1D and F). The solute carrier *slc2a15b* is known to affect leucophore (white pigment cells) development in medaka (Fukamachi et al., 2006; Kimura et al., 2014; Lynn Lamoreux et al., 2005), and is expressed in the xantholeuocophore cells of adult zebrafish (Lewis et al., 2019). Leucophores and xanthophores are thought to be developmentally related (Kimura et al., 2014; Lewis et al., 2019), suggesting that *slc2a15b* is a good candidate for future study. The connexin protein, *gjb8* (previously *cx30.3*), is known to be expressed in the skin, otic, and neural tube of zebrafish (Chang-Chien et al., 2014; Tao et al., 2010). To our knowledge, no functional role for *gjb8* in NCC development has been proposed, but the importance of other gap junction proteins in pigment pattern development in zebrafish (Irion et al., 2014) makes this gene a good candidate for future study.

**Figure 3.**
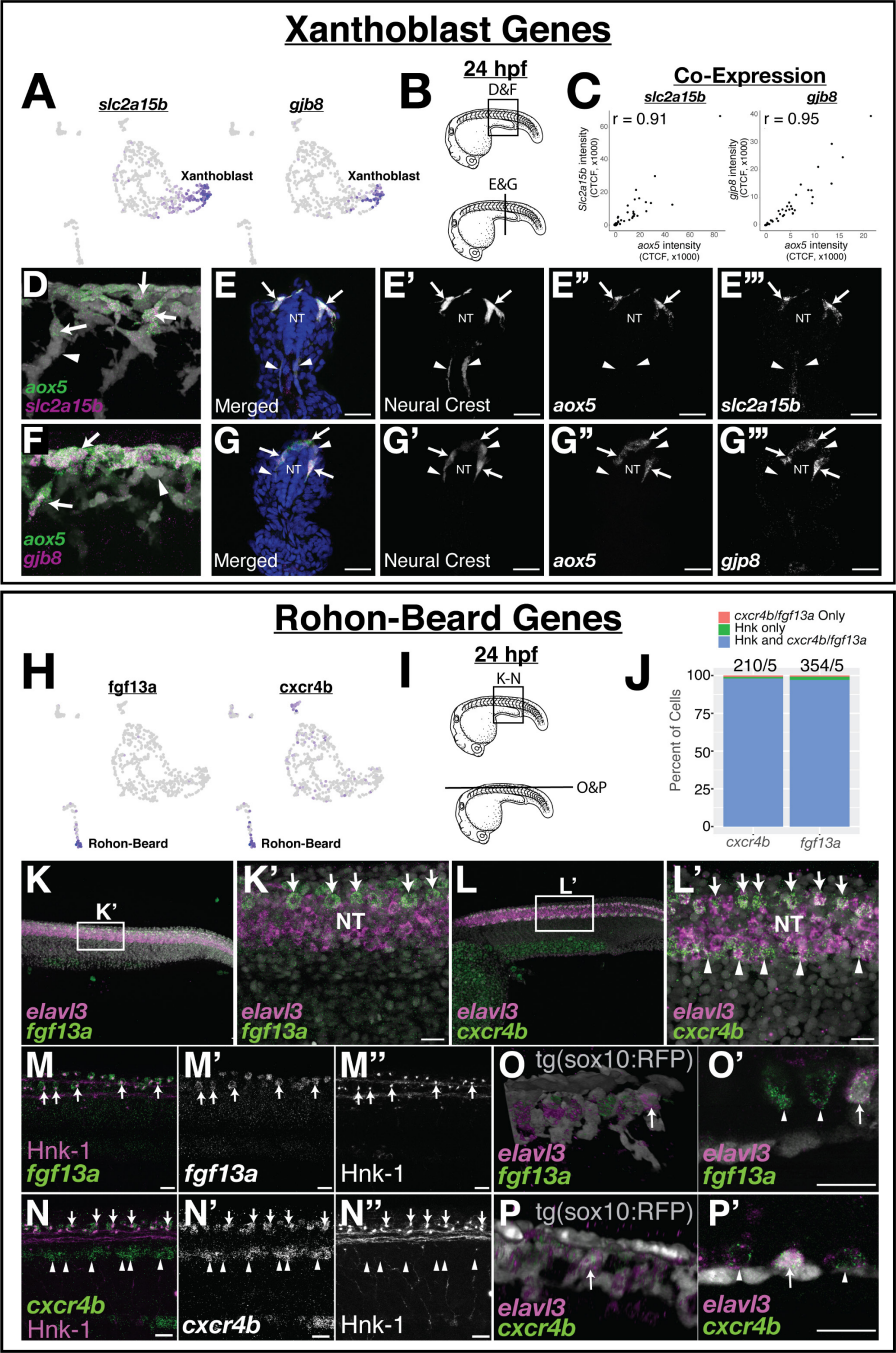
Expression of novel marker gene expression in the pre-migratory xanthoblast and Rohon–Beard cell populations. (**A**) Both *slc2a15b* and *gjb8* are predicted to be expressed in pre-migratory xanthoblast cells. (**B–G**) Quantitative single-molecule in situ hybridization (hybridization chain reaction [HCR]) confirms expression of these genes to *aox5* expressing xanthoblasts. (**B**) Schematic of 24 hr post fertilization (hpf) embryos labeling region of embryos shown in subsequent panels. (**C**) Quantification of RNA expression (corrected total cell fluorescence [CTCF]) in neural crest cells (NCCs) from sections shows novel marker genes *slc2a15b* and *gjb8* are strongly co-expressed with *aox5*. Sections were taken every 15 µM and analyses were limited to sections over the yolk extension. Each dot represents a cell (*slc2a15b* = 3 embryos, 9 slides, 49 cells; *gjb8* = 3 embryos, 9 slides, 58 cells). (**D–G**) Representative whole mount (**D**, **F**) and sections (**E**, **G**) show expression of *slc2a15b* (**D**, **E**) and *gjb8* (**F**, **G**) in 24 hpf embryos. DAPI (blue, **E**, **G**) labels nuclei. Arrows point to NCCs co-expressing xanthoblast marker genes, while arrowheads point to NCCs that lack expression of these same genes. Note that a number of *aox5/slc2a15b/gjb8* NCCs are pre-migratory in the dorsal neural tube (NT). (**H**) Both *fgf13a* and *cxcr4b* are predicted to be expressed in Rohon–Beard neurons (RBs). (**I–P**) HCR confirms expression of these genes to RBs. (**I**) Schematic labeling region of trunk shown in subsequent panels. (**J**) Percent of HNK-1-positive RBs also expressing *cxcr4b* and *fgf13a* indicates that these genes are co-expressed in RB cells. The number of HNK-1, *cxcr4b*, and *fgf13a*-positive cells in the dorsal NT was counted. Note almost all *fgf13a/cxcr4b* expressing cells co-localize to HNK-1-positive RB cells. Sample size reported above bars (#cells/#embryos). (**K, L**) Representative confocal images of whole-mount in situ hybridization for *elavl3*, *fgf13a*, and *cxcr4b* show expression of *fgf13a* and *cxcr4b* as two rows of cells in the dorsal NT (arrows). *Elavl3* marks the NT, and DAPI (gray) labels nuclei. Note ventral NT expression of *cxcr4b* (arrowheads in **L’**). (**M, N**) Dual immunolabeling for HNK-1 and in situ hybridization for *fgf13a* (**M**) or *cxcr4b* (**N**) shows gene expression overlaps with HNK-1 labeling in RBs in the dorsal NT (arrows). Arrowheads mark ventral expression of *cxcr4b* in panel (**N**). (**O, P**) While *fgf13a* and *cxcr4b* expression was never observed in NCCs, a subset of putative RB cells express the tg(*sox10*:tagRFP) transgene (gray in panels **O **and **P**). Shown are whole-mount 3D projections (**O**, **P**) and single slice through z-stack (**O**’, **P’**) showing *fgf13a/cxcr4b/elavl3*-positive cells co-labeled with the *sox10* transgene. Note that *sox10*-positive cells are positioned topologically similar to *sox10*-negative RB cells. Scale bars for all panels are 20 µM. Figure 3—source data 1._Excel file of quantified hybridization chain reaction intensity of *aox5*/*slc2a15b*/*gjb8* cells used to generate plots shown in Figure 3C. Figure 3—source data 2._Excel file of quantified HNK-1/*cxcr4b*/*fgf13a* co-localization used to generate plot in Figure 3J.

To confirm expression of these novel xanthoblast genes to NCCs, we used *aox5* expression to label putative xanthoblasts, and the tg(*sox10*:TagRFP) zebrafish line to mark NCCs. At 24 hpf, we found that both *slc2a15b* and *gjb8* were co-expressed with *aox5* in a subset of tNCCs (Figure 3A–G; Pearson’s *r* = 0.91 and 0.95, respectively). We also observed *gjb8* expression in the skin as previously reported (Tao et al., 2010), and both *gjb8* and *slc2a15b* expression in medial tissue ventral to the neural tube. Within the NC, many of these *aox5/slc2a15b/gjb8* expressing cells were in the dorsal neural tube. We first observe expression of these genes in the NC at the 15–20 somite stage, suggesting that these genes are expressed by NCCs as they become migratory (Figure 3—figure supplement 1). Furthermore, we observed higher expression of *aox5*/*slc2a15b*/*gjb8* in anterior NCCs of 20 somite stage embryos, suggesting that these genes are expressed in an anterior-posterior wave associated with the onset of NCC migration (Figure 3—figure supplement 1). Thus *aox5*, *slc2a15b,* and *gjb8* are co-expressed in pre-migratory and early-migrating tNCCs supporting a hypothesis that tNCCs initiate a xanthoblast GRN prior to migrating from the neural tube.

**Figure 3—figure supplement 1.**
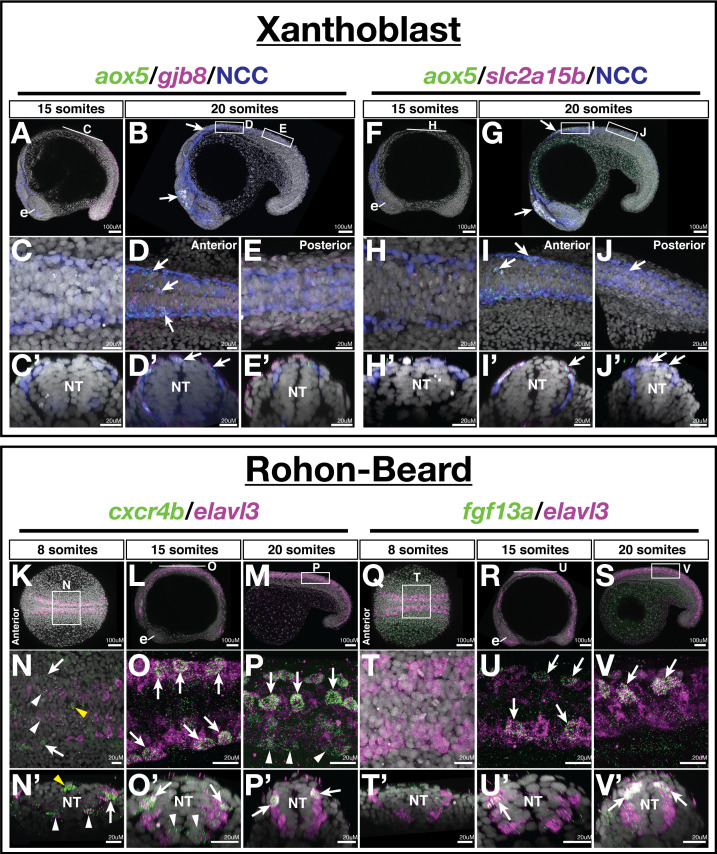
Novel pre-migratory xanthoblast genes and Rohon–Beard (RB) genes are expressed in respective populations as cells differentiate during segmentation stages. Expression of pre-migratory xanthoblast marker genes *gjb8/aox5* (**A–E**) and slc2a15b/aox5 (**F–J**) in 15 somite and 20 somite embryos. Shown are low-magnification whole-mount images (**A, B, F, G**), higher-magnification dorsal views of the neural tube (NT) (**C, D, E, H, I, J**), and associated optical sections of the NT from the same location in the embryo (**C’, D’, E’, H’, I’, J’**). Neural crest cells (NCCs) are labeled with the tg(sox10:TagRFP) transgene (blue), and DAPI (white) marks nuclei. Note that xanthoblast marker genes are weakly expressed in the epithelium of 15 somite embryos and do not co-localize with the sox10 transgene. At 20 somites, xanthoblast marker genes are expressed in a subset of NCCs in both the head and anterior trunk (arrows). NCCs in the posterior trunk express these same genes at lower levels. (**K–V**) Expression of novel RB marker genes *cxcr4b* (**K–P**) and *fgf13a* (**Q–V**) in 8, 15, and 20 somite embryos. Shown are low-magnification whole-mount images (**K, L, M, Q, R, S**), high-magnification views of the NT (**N, O, P, T, U, V**), and associated optical sections (**N’, O’, P’, T’, U’, V’**). Elavl3 expression labels NT, and DAPI (white) labels nuclei in all images. Note that *cxcr4b* is expressed in early differentiating RBs in 8 somite embryos, and differentiated RBs in 15 and 20 somite embryos (arrows). *cxcr4b* expression is also seen in the ventral NT at all stages (white arrowheads), and scattered cells in the dorsal NT of eight somite embryos (yellow arrowheads). In contrast, *fgf13a* is weakly expressed in the epithelium of 8 somite embryos, but strongly expressed in differentiated RBs by 15 and 20 somite stages (arrows). All images are 3D projections, except for optical sections which are max projections of 30–40 slices selected to show expression in relevant tissues. e: eye.

**Figure 3—figure supplement 2.**
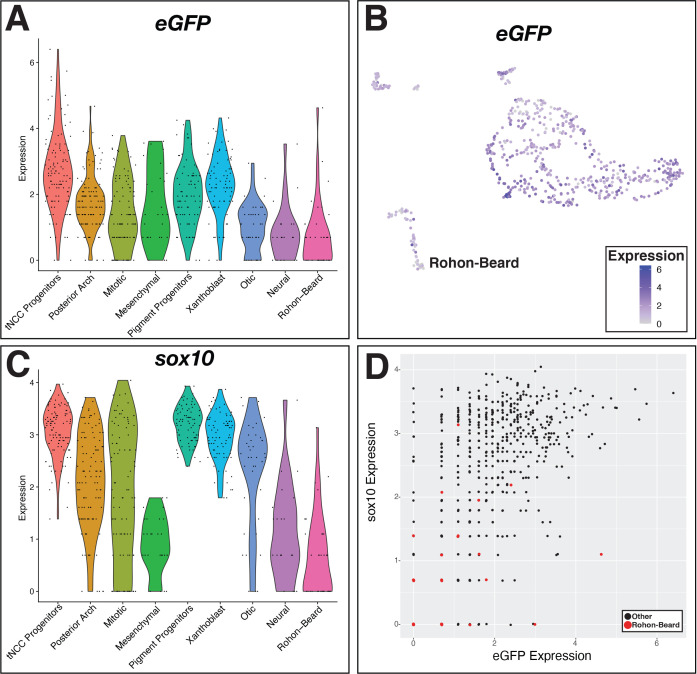
Rohon–Beard (RB) cells express low levels of the tg(*sox10*:eGFP) transgene. Shown are Uniform Manifold Approximation and Projection and violin plots for the predicted expression level of eGFP (**A**, **B**) and sox10 (**C**) transcripts in cells of each cluster. Note the low level of eGFP and *sox10* transcripts predicted to be expressed by RBs. (**D**) Scatterplot shows high eGFP expression correlated with *sox10* expression. RBs (red points) express both genes at low levels.

#### Rohon–Beard neurons express *fgf13a/b* and *cxcr4b*

Our data identify a number of genes not previously known to be expressed by RBs, including non-canonical *fgf13a/b* ligands, the chemokine receptor *cxcr4b*, and the transcription factor pou4f4 (Figure 1—figure supplement 1). Our analyses also found these genes co-expressed in predicted RB cell clusters in independent zebrafish scRNA-seq datasets further supporting these genes as putatively novel RB markers (Figure 1—figure supplement 2). While many novel RB genes appear intriguing, two stand out in particular. Cxcr4b is a chemokine receptor import for cell-cell signaling. Fgf13a/b were among the most differentially expressed RB genes and are non-canonical Fgf ligands that play roles in neuron function and migration, possibly through binding microtubules or voltage-gated sodium channels (Ornitz and Itoh, 2015; Pablo et al., 2016). Thus both genes are candidates for future functional studies.

To confirm the expression of predicted novel RB marker genes, we investigated the expression of *fgf13a* and *cxcr4b* using HCR in situ hybridization. We found that f*gf13a* and *cxcr4b* were expressed in two rows of cells running along the lateral edges of the dorsal neural tube (*elavl3* expression) coincident with the known location of RB neurons (Figure 3F–N). We additionally observed strong *cxcr4b* expression in the ventral neural tube (arrowheads in Figure 3L) and diffuse expression of *fgf13a* in the body. We never observed appreciable levels of *fgf13a*/*cxcr4b* expression in migrating NCCs. To confirm the identity of these *fgf13a/cxcr4b* expressing cells, we labeled RBs with HNK-1 immunofluorescence (Olesnicky et al., 2010). In all embryos, dorsal trunk expression of both *fgf13a* and *cxcr4b* co-localized to HNK-1 labeled cells (Figure 3J, M and N), confirming these *fgf13a/cxcr4b* expressing cells as RBs. We observed expression of these genes in earlier segmentation stage embryos, suggesting that both *fgf13a* and *cxcr4b* are expressed by RBs as these cells differentiate (Figure 3—figure supplement 1). The function of *fgf13* ligands or the *cxcr4b* receptor in RBs is unknown and a focus of future research.

These data raise an intriguing question: why are RBs present in a dataset that used FAC sorting to enrich for NCCs? The answer may be that during our experimentation we occasionally observed RBs labeled with the tg(*sox10*:TagRFP) transgene (Figure 3O–P). In this zebrafish line, these RFP+ RBs were in the same topological position in the neural tube with other RBs, indicating to us that these cells are unlikely to be NCCs. We thus hypothesize that RBs may be represented in our dataset because a small percentage of these cells express the *sox10* transgene similar to NCCs. This hypothesis is supported by low-level expression of both *sox10* and eGFP by RBs in the scRNA-seq data (Figure 3—figure supplement 2). Further, we note that RBs in an independent scRNA-seq dataset (Wagner et al., 2018) also express some NCC genes including *crestin* at low levels (Figure 2—figure supplement 2). These data are intriguing as RBs and NCCs share a developmental origin in the neural plate border (Artinger et al., 1999; Cornell and Eisen, 2000; Fritzsch and Northcutt, 1993; Hernandez-Lagunas et al., 2005), and thus our data are consistent with a literature of developmental similarity between these two cell types.

### Expression of novel xanthophore and RB neuron markers is lost in *Prdm1a* mutants

**Figure 4.**
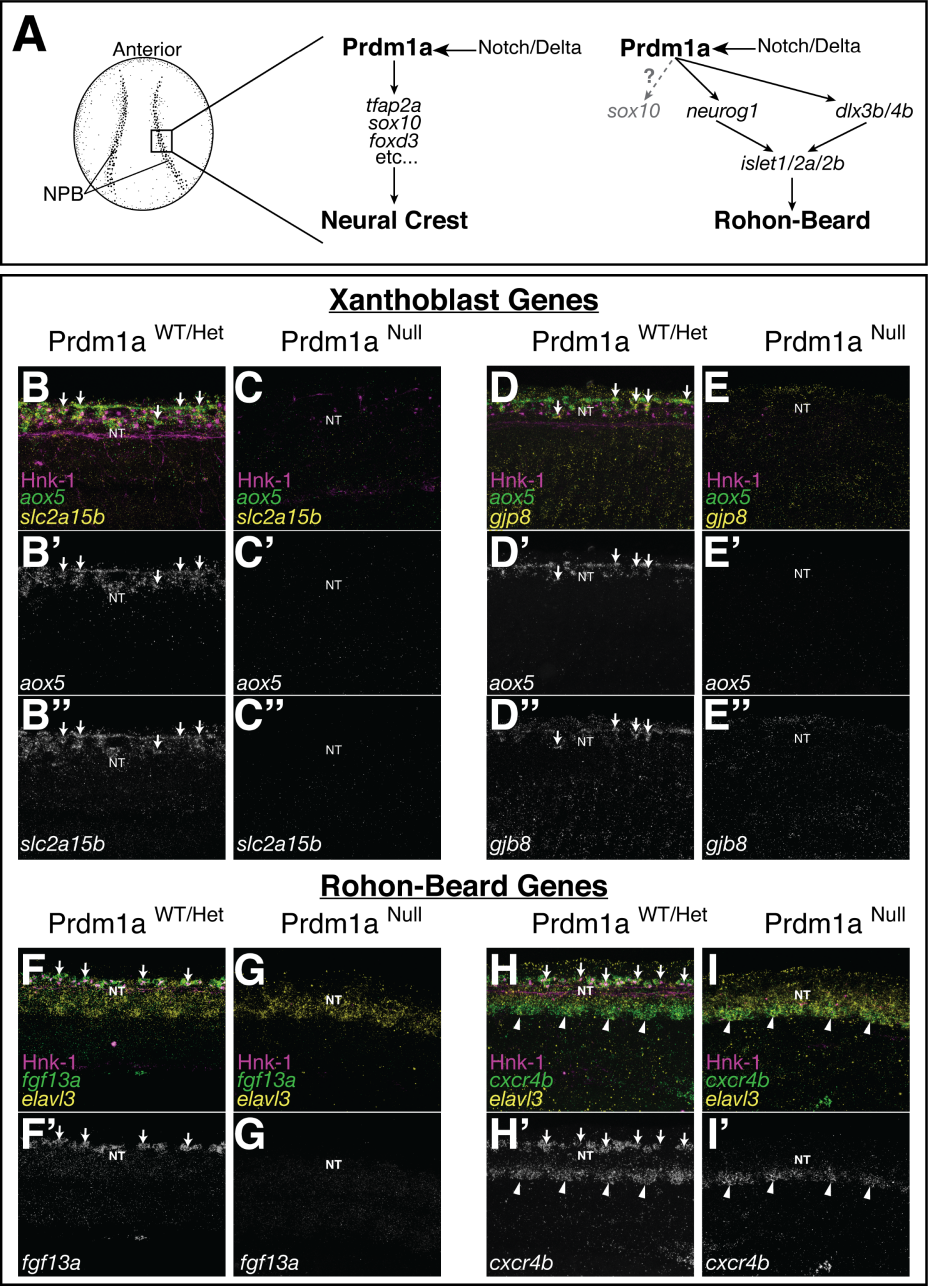
Expression of novel xanthoblast and Rohon–Beard neuron (RB) marker genes is lost in the Prdm1a mutant. (**A**) Schematic of neural crest cell (NCC) and RB specification. Both NCCs and RBs are specified in the neural plate border (NPB) of gastrulating embryos. NCC and RB specification share a similar gene regulatory network (GRN) that includes notch/delta and *prdm1a* expression in the NPB. RBs may transiently express *sox10* at low levels (gray). (**B–H**) Whole-mount confocal images of hybridization chain reaction (HCR) shows expression of xanthoblast (*aox5*, *slc2a15b*, *gjp8*) and RB marker genes (*fgf13a*, *cxcr4b*) in wild type (**B, D, F, H**) and Prdm1a^-/-^ (**C, E, G, I**) embryos at 24 hr post fertilization (hpf). Hnk-1 staining marks RB cells in all images (note loss of HNK-1 in Prdm1a^-/-^ embryos). Expression of all marker genes is lost from the dorsal neural tube (NT) in Prdm1a^-/-^ embryos that show NCC and RB specification defects. Arrows point to expression of marker genes in dorsal neural tube NCCs (**B**, **D**) and RBs (**F**, **H**). Expression of genes in regions of the embryo other than the NC and RB is still present. For example, expression of *cxcr4b* in the ventral neural tube is not altered in the Prdm1a mutant (arrowheads). All images are 3D projections of lateral views of the neural tube taken over the yolk extension of 24 hpf embryos.

Both NCCs and RBs are specified in the neural plate border during gastrulation. Fate determination for both cell types requires expression of the transcription factor *prdm1a* (Figure 4A). Loss of *prdm1a* function leads to loss of both RB and NCC identity (Hernandez-Lagunas et al., 2005; Hernandez-Lagunas et al., 2011; Olesnicky et al., 2010; Powell et al., 2013). To confirm expression of *slc2a15b/gjp8* in NCC-derived xanthoblasts and *fgf13a*/*cxcr4b* in RBs, and to place these genes within known NCC and RB GRNs, we examined the expression of these genes in the NRD/*prdm1a* mutant zebrafish line. Prior studies have shown that *m*utant *prdm1a*^-/-^ fail to specify NCCs and RBs at 24 hpf and have a reduction of melanocytes at 48 hpf (Artinger et al., 1999; Hernandez-Lagunas et al., 2005; Olesnicky et al., 2010). We thus reasoned that if expression of these genes is restricted to NCCs and RBs respectively then we should see loss of expression of these genes in *prdm1a*^-/-^ mutants and this is exactly what we found. Dorsal neural tube expression of *slc2a15b*, *gjp8*, *fgf13a*, and *cxcr4b* were all lost in *prdm1a*^-/-^ embryos (Figure 4B–I). Expression of these genes in areas of the embryo other than NCCs and RBs was not affected (e.g., *cxcr4b* expression in ventral neural tube shown in Figure 4H,I). These data suggest that loss of expression was due to loss of NCC and RB cell specification, confirming expression of these genes to NCCs and RBs and placing these genes downstream of the neural plate border specifier Prdm1a.

### Discussion and conclusion

We found that tNCCs are a transcriptionally heterogeneous population during the early stages of migration and identify a number of previously uncharacterized genes for further study. Our data identify both presumptive multipotent tNCC progenitor cells and a subset of tNCCs already expressing genes associated with differentiated pigment cells prior to migration and at relatively early developmental stages (see also Ling and Sauka-Spengler, 2019; Soldatov et al., 2019). Cells specified towards the xanthophore lineage, in particular, may be one of the earliest tNCC derivatives to begin differentiating.

While we find NCCs expressing markers of differentiated pigment cells, we did not find tNCCs beginning to express genes associated with the PNS. This observation was supported in analyses that compare tNCCs from 24, 48, and 68 hpf embryos (Figure 2). Cells expressing genes associated with PNS are only observed at 48 and 68 hpf when these post-migratory NCCs are already differentiating/differentiated. Thus, while genes associated with pigment cell derivatives are expressed at pre-/early migratory stages, NCCs delay expression of genes associated with PNS cell types until post-migratory stages.

Identification of pre-migratory pigment NCCs is consistent with results from some cell labeling experiments in zebrafish, suggesting that NCCs are lineage restricted prior to migration (Raible and Eisen, 1994; Raible and Eisen, 1996; Schilling and Kimmel, 1994). In these experiments, pre-migratory NCCs labeled in the neural tube only make a single NCC derivative. While these classic cell labeling experiments could not identify whether pre-migratory NCCs were beginning to differentiate, our data extend these studies by showing that some pre-migratory NCCs are already expressing a pigment cell GRN. Whether presumptive pigment cells identified in our scRNA-seq data are truly fate restricted, however, is unknown and would require additional experimental manipulation.

Some scRNA-seq studies have observed multipotent cells transiently expressing genes associated with two or more differentiated fates (Farrell et al., 2018; Soldatov et al., 2019). These data challenge a classical bifurcating model of cell specification, suggesting that instead multipotent cells begin initiating GRNs characteristic of multiple fates before becoming committed to one lineage over others. We do not observe populations of cells expressing multiple fate derivatives in our data, though we do note that xanthoblasts have a more pan-NCC gene expression signature than differentiated xanthophores from 68 hpf embryos. However, we also note that our relatively small sample size for scRNA-seq datasets (607 cells) and lack of multiple sampled developmental stages may prohibit identifying multi-fate expressing cells. Future work sampling earlier stages is likely to provide further insight into how NCC early specification occurs in zebrafish and complement other studies in both mouse and chick (Ling and Sauka-Spengler, 2019; Soldatov et al., 2019; Zalc et al., 2021).

Other studies have used scRNA-seq to identify transcriptionally cryptic populations of cells associated with the leading edge of the cranial NCC migratory front in chick (Morrison et al., 2020; Morrison et al., 2017). In zebrafish, cell tracking studies in the trunk similarly suggest that a subset of migrating tNCCs act as leader cells forging a path along which follower NCCs migrate (Richardson et al., 2016). While we identify multiple transcriptionally unique populations of NCCs in our data, we do not identify a cluster of cells associated with this ‘leader’ cell type. Neither do we find any cluster in our data characterized by expression of orthologous genes that identify trailblazer NCCs in chick (Morrison et al., 2020; Morrison et al., 2017). Thus the identification of zebrafish trunk leader cell transcriptome remains open.

Though our experimental design targeted NCCs, we also sequenced a population of RB neurons and show that in zebrafish some RBs express *sox10* and *sox10* transgenes. While these data do not directly test for a shared progenitor, it is intriguing to consider these data in the context of other studies showing that NCCs and RBs share a similar developmental origin at the neural plate border (Artinger et al., 1999; Hernandez-Lagunas et al., 2005; Olesnicky et al., 2010). Cells at the neural plate border are thought to be an equivalence group, able to differentiate into both NCCs and RBs (Cornell and Eisen, 2002). Interactions among neural plate border cells, mediated in part by notch signaling, bias cells towards one fate over the other (Appel and Eisen, 1998; Cornell and Eisen, 2000; Cornell and Eisen, 2002). Our data suggesting that RBs express both *sox10* and *sox10* transgenes seems to be in line with this idea of a shared developmental potential.

To what extent NCCs and RBs share a common progenitor and whether the evolution of these two tissues is temporally coincident at the root of the vertebrate phylogeny are questions that have long captured the imagination of scholars of vertebrate biology (Artinger et al., 1999; Bone, 1960; Cornell and Eisen, 2000; Fritzsch and Northcutt, 1993). The neural crest is a novel migratory cell population unique to vertebrates with no known homology to tissues in non-vertebrate chordates (Gans and Northcutt, 1983), though see Jeffery et al., 2008; Jeffery et al., 2004. RBs are a non-migratory neuron with uncertain homology to morphologically similar motor neurons in non-vertebrate chordates (Baker and Bronner-Fraser, 1997; Bone, 1960; Gans and Northcutt, 1983). Here, we identify a zebrafish RB transcriptome providing unique insight into the RB GRN. Future work identifying whether orthologous genes are expressed by motor neurons in non-vertebrate chordates could provide further insight into whether these morphologically similar neurons are homologous or homoplastic to vertebrate RB neurons (McCune and Schimenti, 2012; Wagner, 2007). Whether emerging technologies like scRNA-seq will help illuminate the evolutionary origins of new tissue types such as RBs and NCCs only time will tell.

Ultimately, our data suggest that pre-migratory tNCCs are transcriptionally heterogenous and that some pre-migratory NCCs express differentiated pigment cell GRNs. Importantly, this finding is concordant with results from other recent studies in mouse and chick that similarly find NCCs expressing differentiation programs at pre-/early-migratory stages (Ling and Sauka-Spengler, 2019; Soldatov et al., 2019; Zalc et al., 2021). Taken together, these single-cell genomic studies are challenging some classic models of NCC development that emphasize the role of environmental signals from the migratory environment in biasing NCCs towards one derivative state over another (Bronner-Fraser and Fraser, 1988; Bronner-Fraser and Fraser, 1989; Collazo et al., 1993; Trainor and Krumlauf, 2000). In contrast, this emerging work emphasizes interactions among NCC progenitors in the neural tube as critical for specifying at least some NCCs towards different fates prior to migration (Raible and Eisen, 1996).

What mechanisms may be driving these data are speculative and an area of active interest in the field. Notch signaling plays a role in early NCC specification at the neural plate border (Cornell and Eisen, 2000; Cornell and Eisen, 2002), but whether notch signaling continues to play a role in specifying pre-migratory NCCs to different fates at later time points is unknown. Furthermore, there is a temporal component to NCC development. In most taxa, PNS NCCs migrate first and there is a delay before pigment NCC migration occurs. Thus, differences in signals that pre-migratory NCCs receive in the neural tube over time may play a role in biasing cells towards different fates. Ultimately, our and others’ data suggest that unknown interactions in the neural tube are critical for understanding NCC development, and that this pre-migratory period represents an area that deserves continued research attention.

### Materials and methods

**Key resources table:** 

Reagent type (species) or resource	Designation	Source or reference	Identifiers	Additional information
Gene (*Danio rerio*)	Prdm1a	GenBank	ZFIN:ZDB-GENE-030131-2193, Gene ID: 323473	
Strain, strain background (*D. rerio*)	AB	ZIRC	ZDB-GENO-960809-7, RRID:ZIRC_ZL1	
Genetic reagent (*D. rerio*)	tg(–4.9sox10:eGFP)^ba2^	Carney et al., 2006	RRID:ZFIN_ZDB-ALT-050913-4	
Genetic reagent (*D. rerio*)	Tg(sox10:TagRFP)^co26^	Blasky et al., 2014	RRID:ZFIN_ZDB-GENO-150316-2	
Genetic reagent (*D. rerio*)	Prdm1a^m805^	Artinger et al., 1999	RRID:ZFIN_ZDB-ALT-980621-8	
Antibody	Anti HNK-1/N-CAM(mouse, monoclonal)	Sigma-Aldrich	Cat#: C0678, RRID:AB_1078473	IF:(1:20)
Sequence-based reagent	Aox5 RNA Probe	Molecular Instruments	Custom	WM ISH(2 µL/500 µL)
Sequence-based reagent	Slc2a15b RNA Probe	Molecular Instruments	Custom	WM ISH(2 µL/500 µL)
Sequence-based reagent	Gjb8 RNA Probe	Molecular Instruments	Custom	WM ISH(2 µL/500 µL)
Sequence-based reagent	Elavl3 RNA Probe	Molecular Instruments	Custom	WM ISH(2 µL/500 µL)
Sequence-based reagent	Fgf13a RNA Probe	Molecular Instruments	Custom	WM ISH(2 µL/500 µL)
Sequence-based reagent	Cxcr4b RNA Probe	Molecular Instruments	Custom	WM ISH(2 µL/500 µL)
Chemical compound, drug	Accumax	Innovative Cell Technologies	Cat# AM105-500	
Software, algorithm	Velocyto.R (v0.6)	Bergen et al., 2020	RRID:SCR_018167	R implementation
Software, algorithm	Velocyto.py (v0.17.17)	Bergen et al., 2020	RRID:SCR_018167	Python implementation
Software, algorithm	Seurat (v4.0.1)	Butler et al., 2018	RRID:SCR_007322	R implementation
Software, algorithm	Code for analyses	Current study		Source data file: Figure 1&2_RCode.txt
Software, algorithm	R (v4.0.3)	CRAN	RRID:SCR_003005	
Software, algorithm	RStudio	http://www.rstudio.com/	RRID:SCR_000432	
Software, algorithm	CellRanger (v3.2 & v5.0.1)	10X Genomics	RRID:SCR_017344	
Software, algorithm	Fiji	http://fiji.sc	RRID:SCR_002285	
Software, algorithm	Napari	https://napari.org/		

#### Zebrafish lines and husbandry

All zebrafish lines were maintained at the University of Colorado Denver | Anschutz medical school under common zebrafish husbandry conditions. Lines have been previously described and include the tg(*sox10*:eGFP)^ba2^ (Carney et al., 2006), tg(*sox10*:TagRFP)^co26TG^ (Blasky et al., 2014), and NRD^m805^/*prdm1a* mutant line (Hernandez-Lagunas et al., 2005).

##### Single-cell RNA sequencing

Fertilized zebrafish eggs were collected in the morning, stage matched, and reared at 30°C overnight until embryos were at approximately the 25 segment stage corresponding to embryos of ~20–24 hpf at 28.5°C. GFP-positive embryos were dissected posterior to the otic organ using forceps in cold 1x PBS. Trunks from 80 dissected embryos were pooled for dissociation and FAC sorting.

Dissected trunks were dissociated in Accumax (Innovative Cell Technologies) plus DNaseI (1 µL/100 µL). Cells were triturated every 15 min using decreasing-sized pipette tips until a homogenous solution was observed. Cells were filtered through a 40 µM mesh, spun, and resuspended in Sorting buffer (1% FBS/1 mM EDTA/25 mM HEPES in 1× Dullbecco’s PBS; Sigma D8537;). GFP-positive cells were FAC sorted on a MoFlow XDP100 cell sorter into sorting buffer and diluted to approximately 700–1100 cells/µL.

Cells were captured and prepared for sequencing using the 10X Genomics platform by the University of Colorado Denver | Anschutz Genomics Core by loading approximately 10,000 cells. Libraries were sequenced on two lanes of an Illumina NovaSEQ 6000 to an average depth of 159,520 reads per cell and a median of 666 genes per cell. After filtering and quality control, we obtained cells expressing a median of 4255 UMI and 32,425 genes.

##### Single-cell analysis

Reads were mapped to the Ensembl build of the zebrafish genome (GRCz11) and UMI barcodes were assigned to unique identifiers using CellRanger (v3.2). Primary analyses were conducted using the Seurat package v4.0.1 as implemented in R v4.0.3 (Butler et al., 2018). Cells were filtered for quality based on mitochondrial gene expression (≤6% and ≥0.5%) and number of unique transcripts expressed (≥5000 and ≤16,000) in order to remove low-quality cells as well as any possible doublets. Data was normalized using the sctransform function in Seurat (variable features = 3000). Cells were clustered (resolution = 0.6) and projected with the Uniform Manifold Approximation and Projection (UMAP) method using the first 15 principal component analysis (PCA) axes as implemented in Seurat. To find marker genes associated with cell clusters, we performed differential expression using the Wilcoxon rank-sum test as implemented by the FindAllMarkers command in Seurat (logfc.threshold = 0.5, min.pct = 0.25). Changing cell filtering, clustering, and UMAP projection parameters had minor affects and does not change major results of the study (Figure 1—figure supplement 1).

To further explore mitotic cells in cluster 5, we reanalyzed our data regressing out variation due to cell cycle, mitochondrial expression, and ribosomal expression either in combination (Figure 1—figure supplement 1) or individually (not shown). For these analyses, we first calculated an estimate of cell cycle for each cell with the CellCycleScoring function in Seurat using zebrafish orthologs of cell cycle genes available from the Seurat website. We then regressed out the effects of these variables using the SCTransform function prior to clustering and analysis. The primary difference across different analyses is that most mitotic cells in cluster 5 (Figure 1) group with other posterior arch cells from cluster 4 when cell cycle is removed (Figure 1—figure supplement 1). A smaller number of mitotic cells cluster with tNCC progenitors from cluster 1. Importantly, in all analyses we recover similar clusters of cells and marker genes, indicating that the major conclusions from these data are robust to differences in analysis (Figure 1—figure supplement 1).

We used the package Velocyto to infer developmental pseudotime from our data (Bergen et al., 2020). This method uses estimates of RNA splicing from scRNA-seq data to infer how cells may be transitioning across different transcriptional states; the analysis assumes that the ratio of spliced to unspliced transcripts should provide an estimate of the time since a cell has started expressing a new set of genes associated with a different transcriptional state. We used the Python implementation of Velocyto to infer RNA splicing with default parameters, and the R implementation of Velocyto to infer RNA velocity (e.g., pseudo-time) with default parameters and plot velocities onto UMAP space.

To quantify GFP expression in RB cells, reads were aligned to the *Aequorea victoria* green fluorescence protein sequence (EBI accession #AAA27722.1) using cell ranger (v5.0.1). The same analysis parameters as above were used to generate plots.

##### Dataset integration

We integrated our scRNA-seq data with complementary scRNA-seq atlases from Howard et al., 2021 and Wagner et al., 2018. For Howard et al., 2021, we remapped sequences downloaded from SRA (BioProject PRJNA640816) to the Ensembl build of the zebrafish genome (GRCz11) using CellRanger v5.0.1. Cell annotations were downloaded from UCSC Cell browser (cells.ucsc.edu). Cells were filtered to only include those used in the original Howard et al., 2021 study. For Wagner et al., 2018, data and annotations were downloaded directly from NCBI GEO (GSE112294). We only analyze data obtained from 14, 18, and 24 hpf embryos from Wagner et al., 2018.

Datasets were integrated using Seurat IntegrateData function on anchors identified from 2000 genes using log normalized data. We present data integrated with Howard et al., 2021 using the first 30 PCAs for analyses. We present data integrated with Wagner et al., 2018 using the first 100 PCAs for analyses. We get similar results with different parameters when integrating and analyzing the data (not shown). For all analyses, we annotate cells with the original annotations given in Howard et al., 2021 and Wagner et al., 2018, respectively. Differential expression was performed using the Wilcoxon rank-sum test in Seurat on log normalized data.

Code for all analyses is available as source data file: Figure 1—source code 1.

##### In situ hybridizations and immunofluorescence

To confirm expression of genes, we used fluorescent qHCR in situ hybridization (Choi et al., 2018). Custom RNA probes were purchased from Molecular Instruments. Probes were designed using either the B2 or B3 hairpin loop sequences with adaptor fluorophores of either Alexa Fluor 488 (B3) or Alexa Fluor 647 (B2). In situ hybridizations were conducted following Molecular Instruments protocol for whole-mount zebrafish. Samples from 25 somite (24 hpf), 8 somite, 15 somite, and 20 somite embryos were fixed in 4% paraformaldehyde and stored in methanol at –20°C. Samples were rehydrated in phosphate buffered solution (PBS) and permeabilized for 5 min in 1 µg/mL proteinase K at room temperature. Hybridization was conducted using RNA probes diluted to 4 picamols/mL at 37°C overnight. Amplification was performed diluting hairpins to 60 picamols/mL at room temperature overnight.

Following in situ hybridization, Rohon–Beard cells were identified by HNK-1 immunofluorescence. Samples were blocked in IF blocking buffer (2% normal goat serum, 2% BSA, 1× PBS) at room temperature for a minimum of 1 hr. Primary mouse anti-HNK-1 antibody (Sigma) was diluted 1:20 in blocking buffer, and samples were incubated in this primary antibody at 4°C for 24–48 hr (Hernandez-Lagunas et al., 2011), and visualized using secondary goat anti-mouse IgM conjugated to Alexa Fluor 594.

Samples were imaged on a Leica SP8 confocal microscope. For whole-mount images, samples were mounted in 0.2% agarose and imaged at either 10× or 40× magnification. For co-expression of xanthophore genes, samples were cryosectioned every 15 µM and imaged at 63× magnification. For both whole-mount and sectioned samples, we imaged over the yolk extension in order to make images comparable.

Unless noted, all images are 3D projections from either whole-mount or sectioned material produced using either ImageJ or Napari. Image quantification was performed using ImageJ. To calculate co-expression of *aox5* with *slc2a15b* and *gjb8,* we calculated corrected total cell fluorescence (CTCF) from 15 µM sections. Briefly, RNA expression by a cell is proportional to relative fluorescence intensity of the HCR in situ signal within a cell (Choi et al., 2018). NCCs were manually segmented in three dimensions using the segmentation editor in ImageJ based on the expression of the tg(*sox10*:TagRFP) transgene. Average cell fluorescence intensity of *aox5*/*slc2a15b*/*gjb8*, and cell volume, was calculated using the ImageJ 3D suite (Ollion et al., 2013). Average background fluorescence intensity was estimated using regions of the embryo where no signal was observed. CTCF was calculated as

CTCF=(Avg. Signal Intensity∗Cell Volume)−(Avg. background Intensity∗Cell Volume)

We noticed that expression of *fgf13a* and *cxcr4b* in the dorsal neural tube was more binary than quantitative. Thus we simply counted the number of HNK-1-positive cells in the dorsal neural tube that were also *fgf13a*/*cxcr4b* positive.

All plots, and the calculation of Pearson’s correlation coefficient, were produced in R (v.4.0.3). A minimum of three embryos were quantified for all analyses. Sample sizes are reported in relevant figure legends and text. Data is available in source data files: Figure 3—source data 1 and Figure 3—source data 2.

## Data Availability

Sequencing data have been deposited in GEO under accession code GSE163907. The following dataset was generated: LencerE PrekerisR ArtingerKB 2020Single cell RNA analysis of trunk neural crest cells in zebrafishNCBI Gene Expression OmnibusGSE163907

## Additional files

Transparent reporting form
