# Supplementary material for: Single-cell RNA analysis identifies pre-migratory neural crest cells expressing markers of differentiated derivatives
Source: eLife. 2021 Aug 16;10:e66078. doi: 10.7554/eLife.66078 (PMC8367380; doi:10.7554/eLife.66078)
Supplement: Transparent reporting form [file elife-66078-transrepform1.docx]

***eLife’s* transparent reporting form**

We encourage authors to provide detailed information *within their submission* to facilitate the interpretation and replication of experiments. Authors can upload supporting documentation to indicate the use of appropriate reporting guidelines for health-related research (see [EQUATOR Network](http://www.equator-network.org/%20)), life science research (see the [BioSharing Information Resource](https://biosharing.org/)), or the [ARRIVE guidelines](http://www.plosbiology.org/article/info:doi/10.1371/journal.pbio.1000412) for reporting work involving animal research. Where applicable, authors should refer to any relevant reporting standards documents in this form.

If you have any questions, please consult our Journal Policies and/or contact us: [editorial@elifesciences.org](mailto:editorial@elifesciences.org).

**Sample-size estimation**

- You should state whether an appropriate sample size was computed when the study was being designed
- You should state the statistical method of sample size computation and any required assumptions
- If no explicit power analysis was used, you should describe how you decided what sample (replicate) size (number) to use

Please outline where this information can be found within the submission (e.g., sections or figure legends), or explain why this information doesn’t apply to your submission:

For single cell sequencing, the trunks of 80 zebrafish larvae were pooled for fluorescence-activated cell sorting in order to capture enough cells for 10x genomics. Further information can be found in the methods. For in situ HCR confirmation, a minimum of three embryos were observed to ensure consistent patterns of expression. Quantification was performed on a minimum of three embryos. Details can be found in methods and relevant figure legends.

**Replicates**

- You should report how often each experiment was performed
- You should include a definition of biological versus technical replication
- The data obtained should be provided and sufficient information should be provided to indicate the number of independent biological and/or technical replicates
- If you encountered any outliers, you should describe how these were handled
- Criteria for exclusion/inclusion of data should be clearly stated
- High-throughput sequence data should be uploaded before submission, with a private link for reviewers provided (these are available from both GEO and ArrayExpress)

Please outline where this information can be found within the submission (e.g., sections or figure legends), or explain why this information doesn’t apply to your submission:

RNA in situ hybridizations were performed a minimum of two times for each probe, and all probes showed consistent patterns of expression across these replicates. Multiple individual embryos were observed for each probe constituting biological replicates, and minimum of three embryos are reported for quantification. Information is found in methods and relevant figure legends.

**Statistical reporting**

- Statistical analysis methods should be described and justified
- Raw data should be presented in figures whenever informative to do so (typically when N per group is less than 10)
- For each experiment, you should identify the statistical tests used, exact values of N, definitions of center, methods of multiple test correction, and dispersion and precision measures (e.g., mean, median, SD, SEM, confidence intervals; and, for the major substantive results, a measure of effect size (e.g., Pearson's r, Cohen's d)
- Report exact p-values wherever possible alongside the summary statistics and 95% confidence intervals. These should be reported for all key questions and not only when the p-value is less than 0.05.

Please outline where this information can be found within the submission (e.g., sections or figure legends), or explain why this information doesn’t apply to your submission:

Analysis of single cell RNA-seq data was performed consistent with established methods using the programs Seurat and Velocyto. Pearson’s correlation was calculated for expression of xanthophore genes in R. More information can be found in methods and relevant sections of the manuscript.

(For large datasets, or papers with a very large number of statistical tests, you may upload a single table file with tests, Ns, etc., with reference to sections in the manuscript.)

**Group allocation**

- Indicate how samples were allocated into experimental groups (in the case of clinical studies, please specify allocation to treatment method); if randomization was used, please also state if restricted randomization was applied
- Indicate if masking was used during group allocation, data collection and/or data analysis

Please outline where this information can be found within the submission (e.g., sections or figure legends), or explain why this information doesn’t apply to your submission:

Group allocation and blinding of samples was not appropriate for the current study.

**Additional data files (“source data”)**

- We encourage you to upload relevant additional data files, such as numerical data that are represented as a graph in a figure, or as a summary table
- Where provided, these should be in the most useful format, and they can be uploaded as “Source data” files linked to a main figure or table
- Include model definition files including the full list of parameters used
- Include code used for data analysis (e.g., R, MatLab)
- Avoid stating that data files are “available upon request”

Please indicate the figures or tables for which source data files have been provided:

Single cell RNA-seq data are deposited and available in GEO GSE163907.

Data for figure 3 (quantification of intensity) are provided as source data files: “Figure 3-Source Data 1_IntensityQuantification_Xanthophores.xlsx” and “Figure 3-Source Data 2_RohonBeardQuantification.xlsx.”

All code used for analyses provided as source data file: Figure 1-Source Code File-R code.txt.
