# Supplementary material for: Single-cell RNA analysis identifies pre-migratory neural crest cells expressing markers of differentiated derivatives
Source: eLife. 2021 Aug 16;10:e66078. doi: 10.7554/eLife.66078 (PMC8367380; doi:10.7554/eLife.66078)
Supplement: Peer review history

# Decision letter

**DOI:** 10.7554/eLife.66078.sa1

**Affiliations:** Washington University School of Medicine United States

Our editorial process produces two outputs: i) public reviews designed to be posted alongside the preprint for the benefit of readers; ii) feedback on the manuscript for the authors, including requests for revisions, shown below. We also include an acceptance summary that explains what the editors found interesting or important about the work.

**Acceptance summary:**

This manuscript investigates specification and differentiation of the neural crest, a migratory population of stem-like cells that contribute to multiple tissues, including the bones of the skull, pigment, and peripheral nervous system. This work presents a single-cell RNAseq dataset from zebrafish trunk neural crest cells during the early stages of migration that identifies the subpopulations of trunk neural crest cells, new genetic markers and a subset of Rohon-Beard neurons. The paper generates a dataset for further investigations and reports expression of differentiated pigment cell markers in the pre-migratory neural crest populations.

**Decision letter after peer review:**

Thank you for submitting your article "Single cell RNA analysis identifies pre-migratory neural crest cells expressing markers of differentiated derivatives" for consideration by *eLife*. Your article has been reviewed by 2 peer reviewers, and the evaluation has been overseen by a Reviewing Editor and Richard White as the Senior Editor. The reviewers have opted to remain anonymous.

The reviewers have discussed their reviews with one another, and the Reviewing Editor who also read the manuscript, has drafted this to help you prepare a revised submission.

Essential revisions:

1. The authors should more clearly state how this study advances their past work that demonstrated the presence of HNK1+ (NV migratory marker) RB neurons in a well-characterized pdrm1 mutant (Hernandez-Lagunas et al., 2014).

2. Reviewers were also concerned that such an analysis performed at a single stage and at a relatively late time point cannot be used to infer common developmental origin or path. This raises questions about the identification of both sox10+ and sox10- RB neurons – both of which are lost in pdrm1 mutant – this finding should be more fully addressed appropriately.

3. Reviewers felt that there is a disconnect between the title and what forms the bulk of the discussion and figures in this paper. The authors should either change the title or put more emphasis on the findings that premigratory NCCs express markers of differentiated derivatives.

4. A stronger attempt should be made to compare/integrate their limited dataset with other more extensive existing datasets (Wagner et al.,2018) to investigate whether markers (fgf13a, cxcr4b) are expressed at earlier time points to help support the authors' hypothesis? Similarly, the authors do not seek to utilize single-cell datasets at later time points obtained using the same transgenic line to verify whether these markers are expressed (Aubrey et al., 2021). Further mining and integration with available datasets would help strengthen the authors' point.

5. When discussing their finding that some premigratory cells already express differentiated genes as a novelty but, the authors should cite studies that have shown this in the neural crest in zebrafish and other models (Soldatov et al., 2019, Ling et al., 2019).

6. The "unknown" cluster 7 described by the authors as a potential new NCC lineage cluster is most likely (authors should verify this) a previously reported mesenchymal cluster expressing a wealth of collagen genesl; this should be verified and rectified.

7. The claim 'Some of 156 cells (Cluster 5) are presumably neural tube tissue' is unclear, as sox10 found in Cluster 5 does not label neural tube. What are the Cluster 5 marker genes; they should be shown as Supp. Figure 1. Cluster 5 also seems to be split into two subclusters. Do different genes mark these regions? The authors should elaborate on the differential split of sox10-expressing and non-expressing cells within the cluster (feature plots in 1F indicate that sox10 is downregulated in the top left portion of this cluster and the RB cluster).

8. One of the primary novelty points emphasized by the authors is the subset of RB neurons. If this is to be confirmed, the authors should perform KO of some of the known marker genes specific to this cell population to show their relevance to RB cell development? What role do these subsets of NCC-RB cells play? While this experimental work is not essential, in the time of CRISPR/Cas9, querying such candidates in F0 generation would significantly strengthen the manuscript.

9. Important technical points: The study lacks sufficient information to verify the data quality and level of rigour in the analysis. For instance, information such as the number of embryos used to get 607 cells should be provided (this is important for defining genetic heterogeneity). What proportion of embryos were 20hpf and 24hpf? How many cells were loaded into the channel? What was the mean number of genes and UMI's were found per cell? How was the analysis performed, what were the parameters/cut-offs used? How many cells are found within each cluster? Furthermore, the data is not always of the highest quality, and figure annotations should be improved. In general, figures need better annotation and precision – indicating developmental stages on HCR images, the section's location, and clarifying the number of sections used for quantification. Also, figure legends should clearly describe annotation detail.

10. Figure 1 Supplement 1 while very visually appealing, it could have a stronger information content. The authors might consider presenting these results in the form of a dot plot (see Seurat function DotPlot), which in addition to conveying the 10 strongest markers of each of these clusters, would also provide information about (a) their expression level and (b) how specific they are to a particular population that might be helpful to readers. Additionally, the authors might consider adding something that presentation about which of those markers are novel, versus which are well established markers of these cell types. This could even be suitable for promotion into main figure 1, as it would add a lot of information content for readers interested in the neural crest and would clarify the novelty of the authors' findings.

11. The presentation and validation of the pair of novel markers for both the xanthophores and Rohon-Beard neurons are very compelling. However, it seems like there is a missed opportunity here to present more of the novel expression findings in a way that is accessible to a broader audience. While it would certainly be unrealistic to validate them all, it seems like it would be very valuable to provide a broader presentation of the convincing novel markers identified in these populations via differential expression as a figure (or figure panel) – perhaps dot plots or some other format.

12. Lines 217-218 "While we cannot rule out that RB neurons are represented in our dataset due to the proximity of RBs and NCCs to each other in the neural tube". Whereas the presentation in Figure 3g, h that sox10::RFP labels some Rohon Beard neurons is convincing, the authors could go one step further – as they point out, the most likely other potential explanation would be that some of these represent doublets, where an RB neuron and another cell ended up in a droplet together perhaps due to imperfect dissociation. A fairly simple analysis that would help further exclude this possibility would be to simply check whether any of the cells identified as RB neurons express markers that are exclusive markers of another cell type in the dataset that is expected to be sox10+. If they do not, then they are unlikely to represent a combination of two cells. (This could be done in fancy ways, but also simply by checking in scatter plots or some other format the expression of the best markers of other clusters in the RB neurons.) It would provide an additional piece of strong evidence that some RB neurons must have expressed sox10 at some point in their developmental history.

13. That some cells prior to migration express markers of pigment cells is convincingly demonstrated. One is left wondering – do the authors think that ALL pigment cells begin to express their markers prior to migration? Are all of the migrating tNCCs that are not expressing cell-type specific markers bound to become PNS derivatives? If there are data from sox10 lines that perhaps estimate the percentage of cells that give rise to these different cell types. The authors could make statements in the Discussion section to help clear this up.

14. The authors note expression of fgf13a in the RB neuron cluster and comment that "FGF and chemokine signaling are critical for proper morphogenesis.." and later "The RBs are apparently sources of and responsive to morphogens important for development. The authors need to reconsider this interpretation of fgf13 expression in RBs, as Fgf13 belongs to the class of intracellular FGF, iFGFs, which are not secreted and have no identified interaction with signaling FGFRs (Ornitz DM, Wires Dev Bio, 2015).

15. Discussion could have more explanation or speculation about why only NCCs expressing pigment cell markers are observed prior to migration.

# Reply: Author response

**DOI:** 10.7554/eLife.66078.sa2

Essential revisions:

1. The authors should more clearly state how this study advances their past work that demonstrated the presence of HNK1+ (NV migratory marker) RB neurons in a well-characterized pdrm1 mutant (Hernandez-Lagunas et al., 2014).

We present a greater discussion of the reasons for studying the expression of select marker genes in the prdm1a mutant zebrafish line. At the stage studied in zebrafish, HNK-1 marks Rohon-Beard neurons, and unlike chick, does not label migratory NCCs.

Both NCCs and RBs require prdm1a for proper specification. We present a new panel to Figure 4 that summarizes the GRNs for both NCCs and RBs. Characterizing the expression of these genes in the prdm1a mutant is important because it both (1) confirms expression of these genes to the NCC/RB lineages respectively, and (2) puts these genes within known NCC/RB GRNs. We discuss how these data affect our previous and ongoing work in the results (lines: 301-317). These data identify an RB transcriptome and we discuss relevance of this for past and future work in the discussion (lines:362-387).

2. Reviewers were also concerned that such an analysis performed at a single stage and at a relatively late time point cannot be used to infer common developmental origin or path. This raises questions about the identification of both sox10+ and sox10- RB neurons – both of which are lost in pdrm1 mutant – this finding should be more fully addressed appropriately.

We absolutely agree! We do not think that these data directly support a common developmental origin. However, we do not think it is appropriate to ignore the decades of literature hypothesizing a common developmental origin. Finding RBs in our dataset was unexpected result. We were also shocked to observe low level expression of sox10 in RBs. We further note that crestin, another gene that labels NCCs in zebrafish, is expressed by RBs in an independent dataset (Wagner et al. 2018). We have gone through our manuscript to be sure that we are careful in how we address this issue. We feel that it is important to consider these data within the context of other similarities among these two cell populations without making a direct claim of shared developmental origin. We agree that future work is needed to tease this apart. We are currently studying the function and expression of RB GRNs identified from these data, and future scRNAseq analyses at earlier developmental stages may help to further illuminate this long-standing hypothesis.

3. Reviewers felt that there is a disconnect between the title and what forms the bulk of the discussion and figures in this paper. The authors should either change the title or put more emphasis on the findings that premigratory NCCs express markers of differentiated derivatives.

We feel that the additional presentation of data, and inclusion of new analyses comparing our dataset to other scRNA-seq datasets in zebrafish, adds emphasis that makes our title appropriate. In particular, analyses integrating our dataset with those of Howard et al. 2021 and Wagner et al. 2018 support our finding that pre-migratory tNCCs express markers of differentiated derivatives (see below). Thus we feel that our manuscript stresses a finding that pre/early migratory tNCCs are already expressing markers of differentiated derivatives. This is shown by both scRNA-seq analyses and confirmation of some xanthophore marker genes by ISH. While we are willing to change our title if requested, we would rather not. In our opinion the current title communicates a major finding from the data that will be interest to other researchers in the field.

4. A stronger attempt should be made to compare/integrate their limited dataset with other more extensive existing datasets (Wagner et al., 2018) to investigate whether markers (fgf13a, cxcr4b) are expressed at earlier time points to help support the authors' hypothesis? Similarly, the authors do not seek to utilize single-cell datasets at later time points obtained using the same transgenic line to verify whether these markers are expressed (Aubrey et al., 2021). Further mining and integration with available datasets would help strengthen the authors' point.

We think this is a great idea and thus we have integrated our data with both Howard et al. 2021 and Wagner et al. 2018, and we add a detailed discussion of these analyses to the manuscript. This work has led to a new section in the main text, and we have modified our figures to produce a new figure 2 that covers this integrated data analysis.

Importantly, these analyses of the integrated data further support the main findings and arguments that we made in the original manuscript. NCCs cluster by cell identity when integrating our data with Howard et al. 2021 or Wanger et al. 2018. As noted in the comment, Aubrey Howard et al. 2021 samples tNCCs using the same transgene as our own study, but at older developmental stages. We find that cells from our dataset cluster with similar cell types from these older embryos. Pigment cells that we identify as expressing markers of differentiated derivatives group with pigment cells at these later stages. Further, pre-migratory xanthoblasts that we characterize from 24 hpf embryos express the same genes as differentiated xanthophores sampled from 68hpf embryos. Thus, this integrated analysis supports our hypothesis that pre-migratory NCCs are already expressing markers of differentiated derivatives. We do observe some pigment cell maturation and comment on these findings in the manuscript. We note that tNCCs at 24hpf do not express markers of differentiated neurons supporting the hypothesis that NCCs delay expressing PNS associated genes until post-migratory stages.

Integrating our data with Wagner et al. 2018 produces a similar result in that cells in our dataset cluster with similarly annotated cells in the Wagner et al. dataset. Importantly, Wagner et al. sampled whole embryos and annotated a small number of cells as RBs. RBs from our dataset cluster with RBs from Wagner et al. and these cells, derived from these independent studies, express the same genes. These data confirm our own analyses and further extend support for identification of genes previously unknown to be expressed by RBs.

In addition to these integrated analyses, we directly looked at how gene expression changes across developmental time by using HCR to examine expression of xanthoblast and RB marker genes at earlier developmental stages. These data are included as Figure 3—supplement1, support our previous findings, and are discussed in lines: 253-258 and 282-284.

5. When discussing their finding that some premigratory cells already express differentiated genes as a novelty but, the authors should cite studies that have shown this in the neural crest in zebrafish and other models (Soldatov et al., 2019, Ling et al., 2019).

We apologize for this oversight and have rectified discussion of these missing citations. We thank the reviewers for bringing our attention to this oversight on our part.

6. The "unknown" cluster 7 described by the authors as a potential new NCC lineage cluster is most likely (authors should verify this) a previously reported mesenchymal cluster expressing a wealth of collagen genesl; this should be verified and rectified.

This is a great hypothesis and supported by integrating our data with other datasets that fully sample a ‘mesenchymal’ NCC cell type (see above). In these integrated analyses cells in this cluster group with mesenchymal NCCs from these other datasets. We now label these cells as “mesenchymal”.

7. The claim 'Some of 156 cells (Cluster 5) are presumably neural tube tissue' is unclear, as sox10 found in Cluster 5 does not label neural tube. What are the Cluster 5 marker genes; they should be shown as Supp. Figure 1. Cluster 5 also seems to be split into two subclusters. Do different genes mark these regions? The authors should elaborate on the differential split of sox10-expressing and non-expressing cells within the cluster (feature plots in 1F indicate that sox10 is downregulated in the top left portion of this cluster and the RB cluster).

This is a good point. These cells express many neural markers that are also in the neural plate and neural tube (as well as many neurons). We provide a greater detailed discussion of these cells as a new Figure1—supplement 3. We note that these cells are characterized by the expression of many neural genes from the neural plate and a number of zic transcription factors, as well as lower (but not absent) sox10. Thus these cells may represent ‘early’ NCCs still expressing a number of NPB genes. Some of these marker genes are also expressed by a limited number of NCC progenitors. We refer to these cells as ‘neural’ and note in the main text that they are characterized by expression of multiple neural plate genes (lines: 145-152).

The comment that the cluster is split is in reference to when the data are analyzed by regressing out biologic variation due to cell cycle etc. In this case, some cells that otherwise group with NCCs now cluster with neural cells (see Figure 1—supplement1). These neural/NCC cells are almost certainly NCCs and express NCC marker genes (e.g. sox10 etc) but not neural plate genes (olig3, zic2a, etc). This is a rather minor observation in the data, and does not affect any major conclusions, and thus we do not feel a lengthy discussion is necessary on this topic. Instead, a broader discussion of the analysis is provided in the manuscript where appropriate that shows how the same conclusions are reached regardless of different analysis parameters (Figure 1—supplement 1, lines:107-115, 449-460).

8. One of the primary novelty points emphasized by the authors is the subset of RB neurons. If this is to be confirmed, the authors should perform KO of some of the known marker genes specific to this cell population to show their relevance to RB cell development? What role do these subsets of NCC-RB cells play? While this experimental work is not essential, in the time of CRISPR/Cas9, querying such candidates in F0 generation would significantly strengthen the manuscript.

This is a great idea, and one we are pursuing. However, we agree that this work is not essential for the current manuscript and feel that making CRISPR/Cas9 KOs is outside of the scope of the current study. In our experience, phenotypes from mosaic CRISPR F0s are often not seen in the full knockout F1s. We are thus hesitant to do a study of F0 mosaic embryos. To do these KO studies, we would want to make F1 heterozygous mutants carrying loss of function alleles, and study the function of these genes in the F2+ generations. This would take substantial time to generate these mutants and characterize them sufficiently. So while we absolutely love this idea, and it is a focus of ongoing research, we feel that this is future work and not necessary for the conclusions drawn in the current manuscript.

9. Important technical points: The study lacks sufficient information to verify the data quality and level of rigour in the analysis. For instance, information such as the number of embryos used to get 607 cells should be provided (this is important for defining genetic heterogeneity).

We have provided this information in the methods, results, and figure legends where appropriate. We used the trunks from 80 embryos for cell dissociation (lines:91, 418-420).

What proportion of embryos were 20hpf and 24hpf?

All embryos are of the same stage. We age them as 20-24hpf to account for uncertainty in fertilization time. This is fairly standard practice in zebrafish (see Howard et al. 2021). Further, we note that time is a poor substitute for developmental stage as slight differences in temperature can mean that 24 hpf embryos of very different stages. This is why we also have staged these embryos to the 25 segment stage. We have moved this information into the results from the methods (line 91).

How many cells were loaded into the channel? What was the mean number of genes and UMI's were found per cell? How was the analysis performed, what were the parameters/cut-offs used? How many cells are found within each cluster? Furthermore, the data is not always of the highest quality, and figure annotations should be improved.

These are now provided in the methods where appropriate. We loaded approximated 10000 cells. Median number of UMI = 4255 and genes/features = 32425. Analysis parameters and cutoffs are indicated in methods (lines 436-448). Number of cells per cluster is now indicated in parentheses within the results.

In general, figures need better annotation and precision – indicating developmental stages on HCR images, the section's location, and clarifying the number of sections used for quantification. Also, figure legends should clearly describe annotation detail.

We have updated our figure legends to provide more information. Developmental stage and section location is indicated. Samples sizes for quantification are included in appropriate legends.

10. Figure 1 Supplement 1 while very visually appealing, it could have a stronger information content. The authors might consider presenting these results in the form of a dot plot (see Seurat function DotPlot), which in addition to conveying the 10 strongest markers of each of these clusters, would also provide information about (a) their expression level and (b) how specific they are to a particular population that might be helpful to readers. Additionally, the authors might consider adding something that presentation about which of those markers are novel, versus which are well established markers of these cell types. This could even be suitable for promotion into main figure 1, as it would add a lot of information content for readers interested in the neural crest and would clarify the novelty of the authors' findings.

11. The presentation and validation of the pair of novel markers for both the xanthophores and Rohon-Beard neurons are very compelling. However, it seems like there is a missed opportunity here to present more of the novel expression findings in a way that is accessible to a broader audience. While it would certainly be unrealistic to validate them all, it seems like it would be very valuable to provide a broader presentation of the convincing novel markers identified in these populations via differential expression as a figure (or figure panel) – perhaps dot plots or some other format.

These are great ideas. We have updated figure 1 to provide more information on marker genes associated with different cell clusters. We also show additional feature plots for each cluster to visualize gene expression patterns. This new figure 1 provides much more data for readers interested in what genes mark different cell clusters and how restricted different genes are to these different clusters.

We further provide information in supplements to figure 1 that provide further insight into the genes that mark the xanthoblast, RB, and neural cell clusters. We show the top 80 genes (filtering out SI:#### genes for clarity) that mark each of these clusters. These new supplementary figures provide much more information to the reader on known and novel genes that mark these clusters of cells.

12. Lines 217-218 "While we cannot rule out that RB neurons are represented in our dataset due to the proximity of RBs and NCCs to each other in the neural tube". Whereas the presentation in Figure 3g, h that sox10::RFP labels some Rohon Beard neurons is convincing, the authors could go one step further – as they point out, the most likely other potential explanation would be that some of these represent doublets, where an RB neuron and another cell ended up in a droplet together perhaps due to imperfect dissociation. A fairly simple analysis that would help further exclude this possibility would be to simply check whether any of the cells identified as RB neurons express markers that are exclusive markers of another cell type in the dataset that is expected to be sox10+. If they do not, then they are unlikely to represent a combination of two cells. (This could be done in fancy ways, but also simply by checking in scatter plots or some other format the expression of the best markers of other clusters in the RB neurons.) It would provide an additional piece of strong evidence that some RB neurons must have expressed sox10 at some point in their developmental history.

We like this idea a lot and generated a new supplementary Figure that shows this (Figure 1— supplement 4). We note that (1) FAC sorting gated for single cells, (2) our bioinformatic pipeline filtered cells for quality and doublets (e.g. very high number of genes), and (3) scatterplots showing that RBs do not express genes that mark other NCC populations. The one caveat here is that we see three RB cells that do express mitfa. However, these data overwhelming indicate that RBs are not expressing other NCC genes and thus unlikely to be doublets. Interestingly, we do find low sox10 expression in these RB cells. We also note that independent data from Wagner et al. 2018 observe crestin expression in RBs. These data do not rule out the possibility that RBs are doublets, but we find this explanation unlikely.

13. That some cells prior to migration express markers of pigment cells is convincingly demonstrated. One is left wondering – do the authors think that ALL pigment cells begin to express their markers prior to migration? Are all of the migrating tNCCs that are not expressing cell-type specific markers bound to become PNS derivatives? If there are data from sox10 lines that perhaps estimate the percentage of cells that give rise to these different cell types. The authors could make statements in the Discussion section to help clear this up.

15. Discussion could have more explanation or speculation about why only NCCs expressing pigment cell markers are observed prior to migration.

This is a great point. No, we do not find NCCs at 24hpf expressing markers of the peripheral nervous system. This is further supported by integrating our data with Howard et al. 2021 where differentiated peripheral nervous system neurons are present in 48 and 68 hpf embryos. In these integrated analyses NCCs from 24hpf embryos group with pigment cells from older embryos reflecting expression of pigment lineage genes in these pre-migratory NCCs. In contrast, NCCs from 24hpf embryos do not group with neural derivative cells from these 48 and 68 hpf embryos. This suggests that NCCs do not express genes marking PNS derivatives until post-migratory stages. We have included this as discussion in the relevant locations of the results (lines 177-186) and discussion (lines 326-332).

To our knowledge there is not a clear estimate of the percentage of cells that give rise to these different cell types. However our qualitative observations are that fewer NCC cells migrate along the medial path during the 24 hpf time point than are pre-migratory pigment cells in the neural tube.

14. The authors note expression of fgf13a in the RB neuron cluster and comment that "FGF and chemokine signaling are critical for proper morphogenesis.." and later "The RBs are apparently sources of and responsive to morphogens important for development. The authors need to reconsider this interpretation of fgf13 expression in RBs, as Fgf13 belongs to the class of intracellular FGF, iFGFs, which are not secreted and have no identified interaction with signaling FGFRs (Ornitz DM, Wires Dev Bio, 2015).

This was an oversight on our part and one that we also discovered with further reading while our manuscript was in review. We apologize, and have updated our discussion of these data to account for what is known about these non-canonical fgfs. They seem to play a role in solute carrier binding and critical for neuron function. It is intriguing to us that these fgf13 paralogs are not expressed in all neurons, suggesting some specific role that is required for some neuronal function but not critical for pan-neuronal function.
